# The sonic energy of background music impacts cognitive performances: a behavioral and physiological investigation

**DOI:** 10.1186/s41235-025-00676-9

**Published:** 2025-11-18

**Authors:** Maria Francesca Gigliotti, David Lauret, Yvonne N. Delevoye-Turrell

**Affiliations:** 1 Filboost, Strasbourg, France; 2 Univ. Lille, CNRS, UMR 9193 - SCALab - Sciences Cognitives Et Sciences Affectives, 59000 Lille, France; 3 Institut Universitaire de France (IUF), Paris, France

**Keywords:** Task demands, Cognitive load, Arousal, Musical features, Executive functions

## Abstract

Listening to background music while engaging in mental tasks is a popular habit. Despite the diverse playlists conceived for this purpose, the optimal sonic energy (calming vs. arousing) of a musical excerpt that may benefit cognitive performances remains poorly understood, particularly in relation to the specific demands of the task. To clarify this issue, we asked participants to perform an Attention Network Test and a phonemic Verbal Fluency Task, in silence and while listening to low- and high-arousing unfamiliar musical excerpts. Excerpts sonic energy was determined by musical features analysis, followed by a subjective evaluation of the excerpts’ arousal potential. Behavioral, physiological and subjective measures were collected. Results showed that the presence of both the low- and high-arousing music increased physiological activation and enhanced the pleasure experienced during task execution. Behavioral findings revealed beneficial effects of background music on executive control-related attentional abilities and word production fluidity across time. Finally, participants experienced more cognitive effort during the attentional task with the high-arousing excerpt, while no differences were observed in the Verbal Fluency Task. These findings highlight the importance of tailoring background music sonic energy to the demands of the task in order to mobilize resources and enhance enjoyment without disrupting cognitive performances.

**Supplementary Information:**

The online version contains supplementary material available at 10.1186/s41235-025-00676-9.

## Introduction

In 2016, 80% of 4553 respondents surveyed by Totaljobs reported that listening to music increased their productivity at work by masking external distractions (e.g., ambient noise and conversations) and preventing task-irrelevant thoughts. With the proliferation of streaming platforms and advancements in sound diffusion technology, listening to music while working or studying has become a widespread habit. A plethora of diverse playlist have been designed in response to such a demand. Crucially, some playlists propose complex and stimulating excerpts (e.g., classic music airs), while others propose relaxing and calming compositions (e.g., ambient or atmospheric music). Despite many claims of strong and guaranteed focusing effects, scientific evidence remains scarce concerning the optimal music energy level suited for the execution of a given mental task.

To date, the effects of listening to background music on cognitive performances are overall highly debated, since reported effects are either positive, negative or null (e.g., Drai-Zerbib & Baccino, 2017; Furnham & Strbac, 2002; Lehmann & Seufert, 2017a; Lesiuk, 2005; Mammarella et al., 2007; Nadon et al., 2021; Reynolds et al., 2014). Such mixed results can be first explained by the complexity of background music effects, which depend on multiple factors related to individual differences, music characteristics and task nature (e.g., Cassidy & Macdonald, 2007; Dalton & Behm, 2007; de la Mora Velasco et al., 2023; Furnham & Strbac, 2002; Küssner, 2017). Second, several meta-analyses pointed out a lack of theoretical background as well as a significant heterogeneity of protocols, tasks and musical stimuli in existing studies (Cheah et al., 2022; de la Mora Velasco et al., 2023; Kämpfe et al., 2011; Vasilev et al., 2018). As a consequence, these limits prevent driving solid conclusions about background music effects on cognitive abilities and hinder the building of a cohesive conceptualization of key influencing factors.

### Main theoretical frameworks explaining background music effects

In such fragmented background, two main theoretical approaches can be outlined to explain music’s effects on cognitive performance.

#### The arousal-and-mood and hedonic approaches

According to the *arousal-and-mood hypothesis* (Husain et al., 2002; Schellenberg et al., 2007; Thompson et al., 2001), due to its soothing and stimulating properties (Linnemann et al., 2015; Thoma et al., 2012; van Goethem & Sloboda, 2011), music would help regulate the listeners’ mood and arousal, which would in turn facilitate cognitive performances. For instance, listening to relaxing or high-arousing music was reported to alter participants’ physiological activation and improve the performance at attentional tests performed afterward (Angel et al., 2010; Chitwood, 2018; Ferreri et al., 2013; Husain et al., 2002; Jefferies et al., 2008; Marti-Marca et al., 2020). Music-induced moods were also found to affect performances. Positive moods were found to broaden attention and enhance cognitive flexibility, while negative moods narrowed the attentional focus and strengthened task perseverance (Fredrickson & Branigan, 2005; He et al., 2017; McConnell & Shore, 2011; Nijstad et al., 2010; Ritter & Ferguson, 2017). In a similar perspective, other theorists argued that, since music is a pleasant and rewarding stimulus, listening to it would shift motivation from discipline to gratification (Scott et al., 2024), enhancing task enjoyment and available resources (Husain et al., 2002). Within this framework, music effects can be hence translated as the resultant of a mood induction procedure. Nonetheless, this means that these effects are not specific to music, since it can be replaced by any other mood-changing stimulus. Additionally, the arousal-and-mood hypothesis was formulated to explain the effects of music when played *prior to,* rather than during task execution, neglecting the distraction effects occurring in this latter case.

#### The distraction-conflict approach

The second main theoretical approach accounts for such deleterious effects observed when background music is presented during the task. This approach is rooted in Baron’s *distraction-conflict* (Baron, 1986) and Kahnemann’s *limited cognitive capacities* theories (Kahneman, 1973). Drawing a parallel with the presence of others conceptualized in Baron’s works, Gonzales and Aiello (Gonzalez & Aiello, 2019) suggested that background music would act as a distractor, competing with the ongoing task for attentional resources. This resources conflict would result in increased cognitive load and deteriorated performances (Sweller, 2011), through either capacity interference (excessive consumption of available resources) or structural interference (recruitment of the same neural mechanisms). In response to such attentional resources conflict, the system would react with a state of increased physiological activation (*drive/arousal*), resulting from the overload generated by the attempt to process multiple inputs.

Based on the distraction-conflict approach, Gonzalez and Aiello (2019) went one step further and manipulated task complexity in addition to music saliency to study the interference between music processing and task demands. Results showed that music saliency (manipulated by varying volume and number of instrumental layers) improved simple vigilance task performances, but hindered the performances in the complex word pairs association task. As an explanation, authors argued that simple tasks are usually under-stimulating and under-demanding (Levinson et al., 2012), leaving more attentional resources available. Salient background music would hence attract part of these leftover resources, narrowing the attentional focus and preventing mind-wandering. On the contrary, high-demanding difficult tasks would be impaired by the presence of salient background music, which would encroach on available resources and reduce those required for the task at hand (Gonzalez & Aiello, 2019). Coherently with this theoretical framework, other authors evoked also a potential “seductive detail effect” of music (Moreno & Mayer, 2000; Rey, 2012) to pinpoint the tendency of some musical elements to captivate listeners’ attention and impose an additional burden on working memory. For the same reason, the negative effects of background music containing lyrics were imputed to an interference between the language processes involved in the processing of the lyrics and the ones required by verbal tasks (e.g., reading or writing; Avila et al., 2012).

### Current limitations concerning music’s arousing effects

#### Distinguishing distraction-induced from sonic energy-induced arousal

Despite providing solid evidence for the interaction between music, available cognitive resources and task demands, the studies cited so far leave out of the equation a crucial dimension of background music: its inherent arousing potential. Researches grounded in the arousal-and-mood approach have primarily examined music as a mood-altering stimulus before a cognitive task, neglecting the distraction effects occurring when music is presented during the task (e.g., Husain et al., 2002). Conversely, studies based on the distraction-conflict approach conceptualized music as a distractor. Its effects are discussed within a pure cognitive, resource management-centered framework, and the only arousal response considered is the stress response arising from the attentional conflict (Gonzalez & Aiello, 2019). However, music is not merely a distraction. By virtue of its compositional characteristics, music has also an intrinsic energizing/arousing potential. As such, changes in activation levels may results not only from its distracting consequences, but also because from its intrinsic energy (Vigl et al., 2023). It is therefore important to distinguish the arousing effects driven by the distraction from the ones due to music’s intrinsic *sonic* energy, intended as its arousing potential.

#### How to operationalize music’s arousing potential/sonic energy

The definition of sonic energy is not straightforward as the notion of arousal potential is itself ambiguous (Sander, 2024). In affect theories, arousal is classically defined as the state of physiological activation of an individual (Russell, 1980; Yerkes & Dodson, 1908) which influences cognition, perception and behavior (Storbeck & Clore, 2008). However, in other scientific literature the term has also been extended to the description of a stimulus intensity (McConnell & Shore, 2011; Nguyen & Grahn, 2017; Sloboda & Juslin, 2001; Vieillard et al., 2008). By applying the term arousal to different constructs, the distinction among the stimulus’s physical characteristics (its intrinsic energy, intensity), its potential effect (arousing power) and the state of the person (activated or deactivated) has therefore become blurred.

As a consequence, this gave rise to different operationalization approaches of music’s arousing potential. Some authors focused on the energy individuals perceive in the musical excerpt or on the relaxed or activated state they experience after listening to it (e.g., Jefferies et al., 2008; Kiss & Linnell, 2023; Marti-Marca et al., 2020; Nadon et al., 2021). Despite capturing individual differences in arousal perception, this approach relies on self-reports and does not take into account the compositional features of the musical excerpt. Other authors modulated structural features of the excerpt, such as tempo, volume, number of instruments or musical genre (e.g., Gonzalez & Aiello, 2019; Husain et al., 2002; McConnell & Shore, 2011; Thompson et al., 2012). While this methodological choice allows rigorous comparison of excerpts by changing only one element at the time, we believe it may result in a reductive consideration of music’s energy and arousing potential. For example, some irregular drum patterns can be as arousing as an orchestral opera overture, challenging the pertinence of using single parameters like the number of instruments. Finally, focusing on acoustic parameters such as volume creates a confound between the effects due to the amount of acoustic stimulation and the ones due to the affective response to music. In the present work, we propose to define sonic energy as a multifaceted experience that arises from the interaction between the music’s compositional properties and the listener’s subjective evaluation of its arousing potential.

### Rationale of the study

To summarize, due to theoretical divergencies and consequent methodological heterogeneity, to date, it remains unclear to what extent the sonic energy of background music has positive, energizing effects or conversely, disruptive impacts on cognitive performances, load and physiological activation. To clarify this issue, we conducted a study where participants were asked to perform two cognitive tasks in silence (control condition) and while listening to low- and high-arousing background musical excerpts. In line with the distraction-conflict approach (Gonzalez & Aiello, 2019), we considered the case where music is played during instead of before task execution to assess its effects as a concurrent source of information competing for cognitive resources. Following our definition of sonic energy, the excerpts’ sonic energy level (high/low) was operationalized taking into account both a selection of arousal-related musical parameters and subjective ratings of the music’s arousing potential. To gather a comprehensive understanding of what benefits occur at the expense of which costs, we collected behavioral measures to assess cognitive performance, physiological measures to quantify the induced activation and subjective measures of perceived cognitive load, pleasure and parasite thoughts.

The following hypotheses were formulated. In line with the distraction-conflict and the arousal-and-mood approaches, we expected that:

#### Hypothesis 1

The presence of background music should increase physiological activation compared to silence, with a higher activation induced by the high-arousing musical excerpt compared to the low-arousing excerpt.

Given that music is not only resources consuming, but also resources providing (e.g., Husain et al., 2002; Scott et al., 2024), we expected that:

#### Hypothesis 2

Sonic energy should improve cognitive performance while exerting only a minor impact on perceived cognitive load. Perceived task easiness and pleasure should be increased in the presence of music compared to silence.

Since different tasks elicit different levels of cognitive load (Gonzalez & Aiello, 2019; Kiss & Linnell, 2023) and that background music was found to differently impact linguistic and non-linguistic tasks due to cross-modal interference (Avila et al., 2012; Cheah et al., 2022), we also manipulated task demands. We selected the Attention Network Test and the Verbal Fluency Task, as they mobilize two complementary cognitive functions (spatial attention vs. mental flexibility and language access abilities) implied in everyday tasks performed while listening to music (e.g., writing, coding, see Methods). This allowed to use only two tasks assessing generalizable cognitive processes and to limit the load for the participant. In line with existing literature, we expected that background music sonic energy effects should be modulated by task nature. Specifically:

#### Hypothesis 3a

The attentional task, which we expect to be less stimulating due to its repetitive, reactive and non-verbal nature, should benefit more from high-energy music, which would compensate the under-stimulation and reduce mind-wandering (Gonzalez & Aiello, 2019; Levinson et al., 2012).

#### Hypothesis 3b

On the contrary, the Verbal Fluency Task, which we expect to be more stimulating due to the involvement of mental flexibility, language and production processes, should benefit more from the low-energy music, providing enjoyment while avoiding overstimulation.

## Methods

### Participants

G*Power (version 3.1) estimated a minimum sample size of 28 participants for a within-factors repeated-measures ANOVA (1 group, 3 measurements; 80% power, *α* = 0.05) and a medium effect size (*Cohen’s f* = 0.25), assumed based on the smallest effect size identified in the literature for similar tasks (Marti-Marca et al., 2020; Ransdell & Gilroy, 2001). To account for potential data loss, we included 34 healthy participants (17 males, 17 females, mean age = 24.15, range 18–40). Among them, 23 (68%) identified as non-musicians or music-loving non-musicians, 11 (32%) as amateur or serious amateur musicians and none as professional musicians (following OMSI item 10, Ollen, 2006,1).

Inclusion criteria were: (a) having French as mother tongue (to avoid difficulties in the Verbal Fluency Task); (b) no major hearing or motor dysfunctions (e.g., profound or partial deafness, paralysis); (c) no neurological, psychiatric or neurodevelopmental disorders (e.g., ADHD); (d) no substance use disorders or smoking/vaping habits (to avoid autonomic activity perturbations). As compensation, participants received a €10 gift card and a recording, in the form of a.txt file, of their cardiac and breathing rhythms while listening to a musical excerpt of their choice. Informed consent was provided prior to scheduling the session. The protocol was approved by the ethical committee of the University of Lille (Ref. Number 2023-716-S120) and conducted in compliance with the Declaration of Helsinki (World Medical Association, 2013).

### Materials

#### Musical excerpts

Two positive instrumental, ambient musical excerpts were selected from a pre-validated database composed by Filboost^©^ specifically for the study. They had a repetitive structure, no lyrics and no distracting details to avoid any salience effect induced by music variations (Lehmann & Seufert, 2017b). The low-arousing excerpt (10 min 48 s, C Major, 60 bpm) was characterized by a simple rhythm, a relaxing melody and soft tones. The high-arousing excerpt (10 min 44 s, C Major, 120 bpm) had lively rhythm and melody and brilliant tones. Extremely low and high sonic energy levels were avoided to remain in the optimal arousal range ensuring performance quality (Yerkes & Dodson, 1908). The tempi were chosen according to previous studies (Vieillard et al., 2008), situating the low-arousing tempi between 40 and 75 bpm and the high-arousing tempi above 100 bpm. The 120-bpm tempo was chosen as we considered it to be sufficiently energetic but not over-stimulating (Yerkes & Dodson, 1908). The two excerpts are available at the following link: 10.5281/zenodo.13374906.

The excerpts’ sonic energy was doubly assessed through (1) a musical feature analysis using the MIRToolbox (Lartillot et al., 2008) and (2) a subjective assessment of perceived arousal in a pilot study. Both analyses confirmed the presence of a difference in arousing potential level between the two excerpts (see Supplementary Material for further details). As a complementary check, in the present study, participants were invited at the end of each condition to rate the excerpts in terms of perceived *arousal* (“How did you find the excerpt?” using a 20-point scale ranging from 0—very relaxing—to 20—very stimulating*)* and *valence* (“How did you find the excerpt?” from 0—very negative—to 20—very positive*)*.

#### Behavioral measures

##### Attention Network Test (ANT short version)

**Fig. 1:**
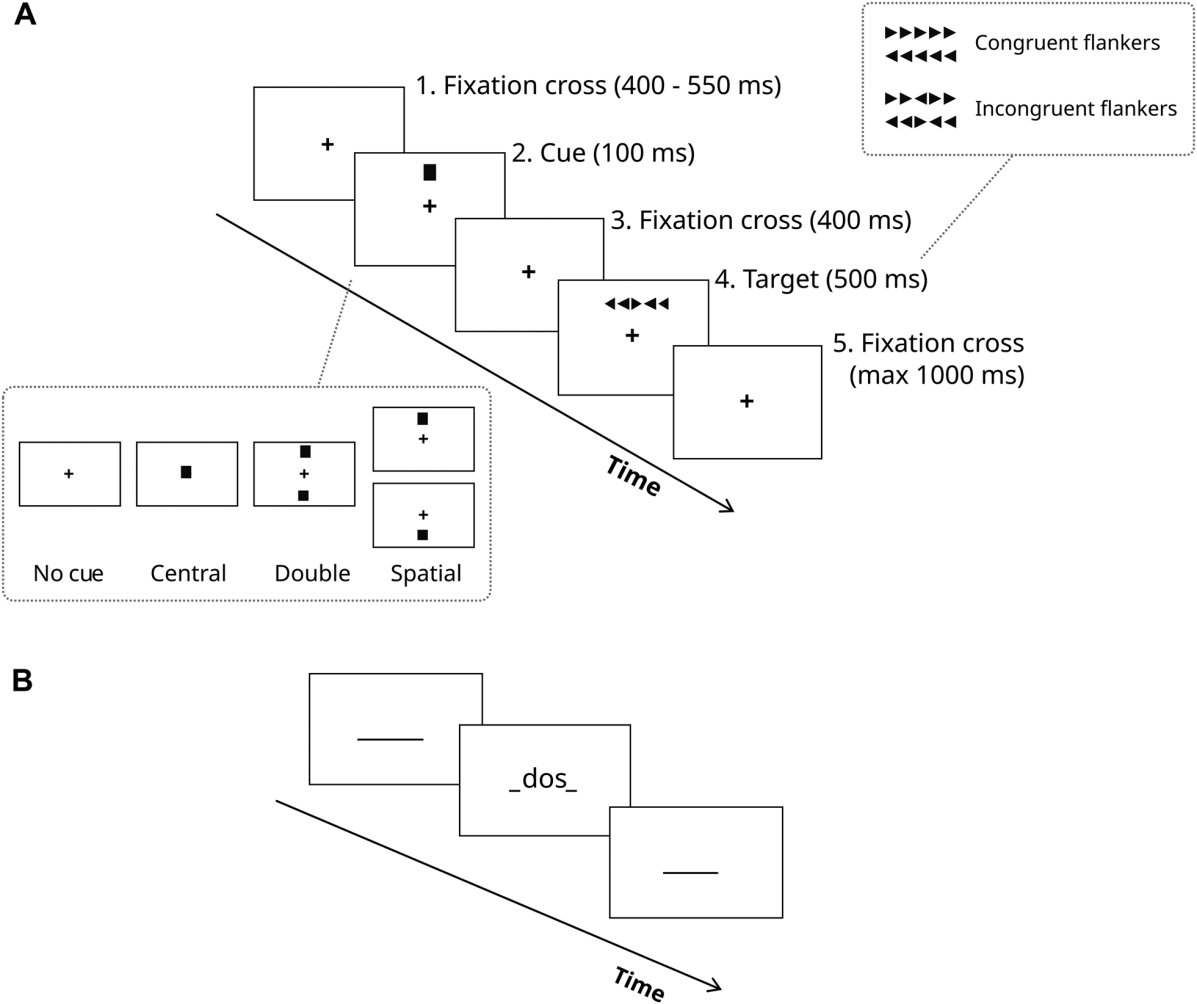
Temporal unfolding of an Attention Network Test and a Verbal Fluency Task trial. *Note*. In the **Attention Network Test** (panel A), participants indicated as quickly and accurately as possible the direction of a target arrow surrounded by congruent or incongruent flankers. The target could be preceded by either an informative or uninformative cue. In the **Verbal Fluency Task** (panel B), participants were presented with an empty textbox and were asked to type a word beginning with the indicated letter. Once validated, the word disappeared, leaving a new empty textbox

Attentional abilities were assessed using the 10-min short version of the ANT (Fan et al., 2002, 2005; Weaver et al., 2013), which evaluates three attentional mechanisms involved in everyday tasks: alert (or phasic alertness, the reactivity to an external warning stimulus), orientation (the shift of attention to a stimulus) and executive control (the detection and processing of conflictual information). The ANT combines a Posner task (Posner et al., 1980) and a Flanker task (Eriksen & Eriksen, 1974). It requires to indicate as fast and accurately as possible the direction of an arrow (target), surrounded by congruent or incongruent flankers (distracting arrows pointing, respectively, in the same or opposite direction of the target). The sequence of arrows could be preceded by the presentation of an informative or uninformative cue (central, double, spatial or absent/no cue). Figure 1A illustrates the temporal unfolding of an ANT trial. All types of cues and flankers were equiprobable and were presented in random order. Participants were given 1500 ms to provide their answer, by clicking on the right or left arrow keys of the computer keyboard with their right index and middle fingers. The sequence of arrows disappeared after 500 ms, but participants could benefit from the remaining 1000 ms to respond. The production of an answer forced the end of the trial. Participants completed 2 blocks of 64 trials (128 trials in total) in each sonic condition (64 trials * 2 blocks * 3 sonic conditions).

##### Verbal Fluency Task (VFT phonemic version)

The VFT assesses mental flexibility (the ability to switch among mental strategies and information) and language access efficiency (Chouiter et al., 2016; Moscovitch, 1994; Schmidt et al., 2017), two processes involved in everyday production tasks such as writing, communicating or brainstorming. Participants completed the *phonemic variant* of the VFT, which required to produce as many words as possible beginning with a given letter within an allotted time (2 min per each sonic condition). The task was executed on a computer. Participants typed each word into a text box (under the form of a horizontal line) and submitted it by pressing the enter key. This action marked the completion of the trial. At the end of a trial, a new empty text box appeared for the next trial (see Fig. 1B). Previous entries were hidden to mimic the task execution conditions of the oral VFT.

Participants were not allowed to write words sharing the same root (e.g., "art" and "artist"), nor proper names of cities, countries, people or brands. To avoid correct spelling retrieval as an interference factor, small accent errors were tolerated provided that they did not impact the global understanding of the word. To avoid learning effects, three different letters were used in a counterbalanced order for each sonic condition: R, D and P. These letters were selected because they provide the greatest number of possible answers in the French language: 11,312 words starting with R, 10,370 words with D and 10,268 words with P. These counts were obtained from the lexique.org website, after filtering out composed words (e.g., "a priori"). To avoid time-consuming retrieval of the orthographic form, vowels were discarded from the selection process, as in the French language they can be associated with different phonemes depending on the vowel they are followed by.

#### Self-reported measures

At the end of both the ANT and VFT, participants completed:

##### The NASA task load index (NASA-TLX)

This questionnaire (French version by Cegarra & Morgado, 2009) assesses the cognitive load experienced during a task through 6 items: mental demands (“*How mentally demanding was the task*?”), physical demands (“*How physically demanding was the task?*”), time pressure (“*How hurried or rushed was the pace of the task?*”), quality of performance (“*How successful were you in accomplishing what you were asked to do?*”), degree of effort (“*How hard did you have to work to accomplish your level of performance?*”) and degree of frustration (“*How insecure, discouraged, irritated, stressed and annoyed were you?*”). Participants responded to each item using a 20-step scale, ranging from 0—*little*—to 20*—a lot*.

##### The subjective experience of task execution

For each sonic condition, participants rated (1) the occurrence of parasite thoughts* (“Did you experience parasite thoughts during the task?”* from 0—*none*—to 20—*a lot)*, (2) the easiness of the task (“*The sonic environment made the execution of the task…:*” from 0—*very difficult*—to 20—*very easy)* and (3) the pleasantness of the task (“*The sonic environment made the execution of the task…:*” using a 20-step scale ranging from 0—*very unpleasant*—to 20—*very pleasant).* The 20-point scales were selected to maintain consistency across all questions and to align with the validated scale format employed in the NASA-TLX.

#### Physiological measures

Physiological responses were collected during the tasks using a BIOPAC device, composed of an MP150 data acquisition module (Model 707A-00008F3), an RSPEC-C module (Model BN-RX) and a universal interface module (UIM100C). The hemodynamic activity was recorded using 3 disposable ECG electrodes connected by a 45-cm triple cable (Model BN-EL45-LEAD3). Two electrodes (references) were placed slightly above the participants’ left and right clavicles; the third one (the ground) was placed on the participants’ left lower abdomen, against the ribs. The respiratory activity was recorded through a wireless breathing belt (Model BN-RESP-XDCR) placed around the chest wall at the level of the sternum. Sampling frequency for both the hemodynamic and respiratory signal recording was set at 1000 Hz. A BioNomadix wireless transmitter (Model BN-TX RSPEC-4.3) mounted on a belt transmitted the data collected by the breathing belt and heart electrodes to the RSPEC-C module.

### Procedure

Two days before the experiment, participants were instructed to abstain from strenuous physical exercise within 24 h (Stanley et al., 2013), acute alcohol consumption within 24 h (McKinney et al., 2012) and stimulant intake (coffee or tea) within 6 h preceding the experimental session (Grant et al., 2018, 2023) to avoid confounding alterations of the autonomic activity. The experiment was conducted in semi-darkness in an experimental box. First, participants were invited to sit in front of the experimental setup and take a few minutes to relax and regain a state of calm. Next, they were equipped with the cardiac electrodes and respiration belt. Lights were then switched off and participants were asked to close their eyes for 3 min, to allow their pupils to adjust to the reduced brightness of the room. Participants proceeded then with a short training on the ANT (32 trials, lasting approximately 2.5 min) and the VFT (1 min). During the ANT training, participants received visual feedback on their performance after each trial (reaction time and accuracy) to ensure correct understanding of the instructions. The training phase was performed in silence. After the training phase, participants performed the ANT followed by the VFT. Task order was kept fixed across participants. Physiological measures were collected during tasks performance. After each task, participants completed the NASA-TLX and rated the subjective experience of performing the task.

**Fig. 2:**
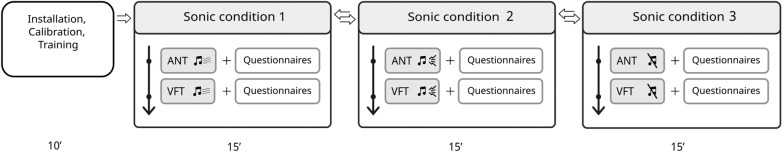
Schematic diagram of the experimental session*. Note.* ANT = Attention Network Test; VFT = Verbal Fluency Task. The three music icons symbolize, respectively, the low-arousing (smooth waves), high-arousing (jagged waves) and silence conditions (crossed-out note). Sonic conditions order was counterbalanced across participants. The musical excerpts were presented only during the execution of the ANT and VFT. Self-reported questionnaires were filled in silence. Hemodynamic and respiratory activities were recorded during the execution of the ANT and VFT, but not during the questionnaires

Figure 2 illustrates the temporal unfolding of the experiment. Tasks and questionnaires were performed 3 times, one per sonic condition: in silence (control), while listening to the low-arousing and the high-arousing excerpt. The order of sonic conditions was counterbalanced across participants using a balanced Latin Square. Musical excerpts were played through a pair of headphones (Beyerdynamic Custom One Pro Plus model, with passive ambient noise-reduction) plugged into the laptop with a jack cable. To avoid an impact of sound onset on the first trials, the musical excerpts were played 8 s before the beginning of each task. They were paused during ANT breaks and stopped at the end of each task. No music was played during the completion of the questionnaires. The experimental session lasted approximately 1 h. Several breaks were planned throughout the experimental session between the blocks and tasks; their duration was tailored to each participant’s needs.

### Data acquisition and preprocessing

The tasks and questionnaires were run on Psychopy (version 2022.1.1, Peirce et al., 2019) using a Dell Precision 3561 laptop (1280 × 1024 screen). All stimuli and texts were presented in white on a gray background to achieve a medium overall screen luminance. Hemodynamic and respiratory activities were collected using the BIOPAC system and Acknowledge software hosted by a second laptop (DELL Latitude E6530). The Psychopy script managed the synchronization between the systems and sent trials-related triggers to the BIOPAC to align trial events to psychophysiological recordings.

ANT performances were evaluated based on reaction times (RT in ms, pooled from correct trials), accuracy and the scores obtained for the three networks: *Alert* = RT_no-cue_–RT_double-cue_, *Orientation* = RT_central-cue_–RT_spatial-cue_ and *Executive control* = RT_incongruent_–RT_congruent_.

VFT performances were evaluated by analyzing the percentage of correct words produced and the delay between each production (in seconds). The inter-word delay was calculated by considering the time elapsed between the press of the enter key (indicating the end of a trial) and the second letter of the following word. The second letter was preferred over the first to counteract the tendency of some participants to write down the first letter before thinking of the full word to write. Incomplete words written at the end of the allotted time were excluded from analyses. Inter-word delays were averaged across participants.

The NASA-TLX questionnaire was analyzed by computing a score out of 20 points for each subscale. A mean global score was also calculated (Raw TLX, for further details on this approach see Byers et al., 1989; Cegarra & Morgado, 2009). Similarly, a score out of 20 points was calculated for the questions on task pleasantness, task easiness and presence of parasite thoughts.

The cardiac and respiratory signals were preprocessed using Python (version 3.1.1.4) and the Neurokit2 package (version 0.2.5; Makowski et al., 2021). Cardiac signals were cleaned using the *neurokit* method provided by the package (0.5 Hz high-pass Butterworth filter [order = 5], followed by powerline filtering [powerline = 50]). Subsequently, they were subjected to interval-related analysis using the function ecg_intervalrelated, to extract mean heart rate (HR) and root mean square of successive differences in heart rate (RMSSD), used to index heart rate variability (Forte et al., 2019; Pham et al., 2021).

Respiratory signals were first downsampled at 50 Hz, then cleaned using the khodadad2018 filtering method provided by the package (Second order 0.05–3 Hz bandpass Butterworth filter) and processed by the rsp_process function (method “khodadad2018”). Mean respiration rate (RR), inspiration/expiration time ratio (I/E Ratio) and coefficient of variation (CV, used to index respiration rate variability, Boiten, 1993; Boiten et al., 1994; Grassmann et al., 2016a; Johannknecht & Kayser, 2022) were extracted using the function rsp_intervalrelated. Autocorrelated variability (AR, indexing respiration variability as well) was calculated at one breath lag (Grassmann et al., 2016a, 2016b; Vlemincx et al., 2011). Table 1 provides a detailed description and interpretation of the physiological indices selected for the analyses.

**Table 1:** Description of the physiological indices analyzed

Index	Description	Underlying physiological phenomenon
*Hemodynamic activity*		
HR	Heart rate: number of heartbeats per minute (bpm)	A fast heart rate indicates a state of physiological activation
RMSSD	Root mean square of successive differences between normal heartbeats (ms)	Most robust index of parasympathetic activity. It highly correlates with HF and pNN50, but contrarily to them, it is less affected by respiration, noise and daily fluctuations. A higher RMSSD indicates a state of calm and relaxation, and a weaker RMSSD a state of physiological activation
*Respiratory activity*		
RR	Respiration rate: number of breaths per minute (br/min)	An increase in respiration rate has been observed in the presence of cognitive load
I/E ratio	Ratio of inspiration to expiration times	An I/E ratio > 1 (i.e., expiration longer than inspiration) might be indicative of a state of physiological activation
CV	Coefficient of variation (%): ratio of the standard deviation to the mean. Here CV is computed on RR	Index of the total respiration variability, which is the sum of random variability (phasic) and autocorrelated variability (AR, tonic). Increased total variability has been observed in case of cognitive load, decreased total variability during sustained psychological states. Changes in CV must be interpreted in relation to changes in AR (Grasman 2016)
AR	Correlated variability (autocorrelation at 1 breath lag)	Index of the tonic variability (the internal stability of respiratory activity). Stronger index of cognitive load than CV, as decreased AR has been systematically observed during challenging tasks. When paired with increased CV, decreased AR suggest that total variability rises due to random variability and externally fluctuations

*Note*. HF = high-frequency band of cardiac signal (0.15–0.40 Hz); pNN50 = percentage of consecutive NN intervals differing by more than 50 ms. Both indices reflect parasympathetic activity

### Statistical analysis

Statistical analyses were performed with R (version 4.2.2, R Core Team, 2022) and R-Studio (version 2022.07.2 + 576). Before the main statistical analyses, outliers were removed using the median absolute deviation (MAD) method (Leys et al., 2013). The rejection threshold was set at the median ± 2 times the MAD. For the hemodynamic and respiration activities datasets, consisting of a unique data point per condition, outliers were winsorized (replacement by the nearest acceptable value) to avoid missing data. Parametric Repeated-Measures (RM) ANOVAs were carried out using the function anova_test of the package rstatix (version 0.7.0). Simple effects and pairwise comparisons (Holm-Bonferroni p-values adjustment) were conducted using the function pairwise_t_test of the same package. Normality was checked by visual inspection of the Q-Q plots and the Shapiro–Wilk test. In case of a major violation of the normality assumption, alternative nonparametric one-way (Friedman RM ANOVA, *χ*^*2*^), two-way and three-way tests (Rank-based RM ANOVA, *F*) and pairwise comparisons (Wilcoxon’s paired signed-rank test, *Z*) were carried out. Greenhouse–Geisser corrections were applied in case Mauchly’s test revealed a violation of the sphericity assumption. Significance levels were set at *α* < .05 for hypothesis testing and at *α* < .10 for normality and sphericity testing.

### Transparency and openness

The study follows APA Style Journal Article Reporting Standards. It reports how we determined our sample size as well as all measures, data exclusions and manipulations realized. Musical excerpts, data and analysis codes are available at 10.5281/zenodo.13374906. This study’s design and its analysis were not pre-registered.

## Results

Below are reported the results directly addressing the research hypotheses. Complete results can be found in the Supplementary Material along with detailed descriptive statistics.

### Musical excerpts evaluation

In line with the pilot subjective assessment study and the musical feature analysis (see Supplementary Analysis 1), the sonic energy of both excerpts was correctly perceived by participants. Arousal potential rating fell significantly below the scale midpoint of 10 for the low-arousing musical excerpt (*M* = 5.53; *t*_*(33)*_ = − 6.68, *p* < .001, *d* = − 1.15), but was significantly above 10 for the high-arousing musical excerpt (*M* = 14.09; *t*_*(33)*_ = 8.72, *p* < .001, *d* = 1.50). Both musical excerpts were perceived as globally positive (high arousing: *M* = 13.68; *t*_*(33)*_ = 5.75, *p* < .001, *d* = .99; low arousing: *M* = 15.91; *t*_*(33)*_ = 9.25, *p* < .001, *d* = 1.59).

### Psychophysiological measures

All hemodynamic and respiratory indices were analyzed through 2-way RM ANOVAs (Sonic Condition [silence, low arousing, high arousing] × Task [ANT, VFT]). Supplementary Table S3 reports mean and standard deviation values for each index.

#### Hemodynamic activity

##### Heart rate

**Fig. 3:**
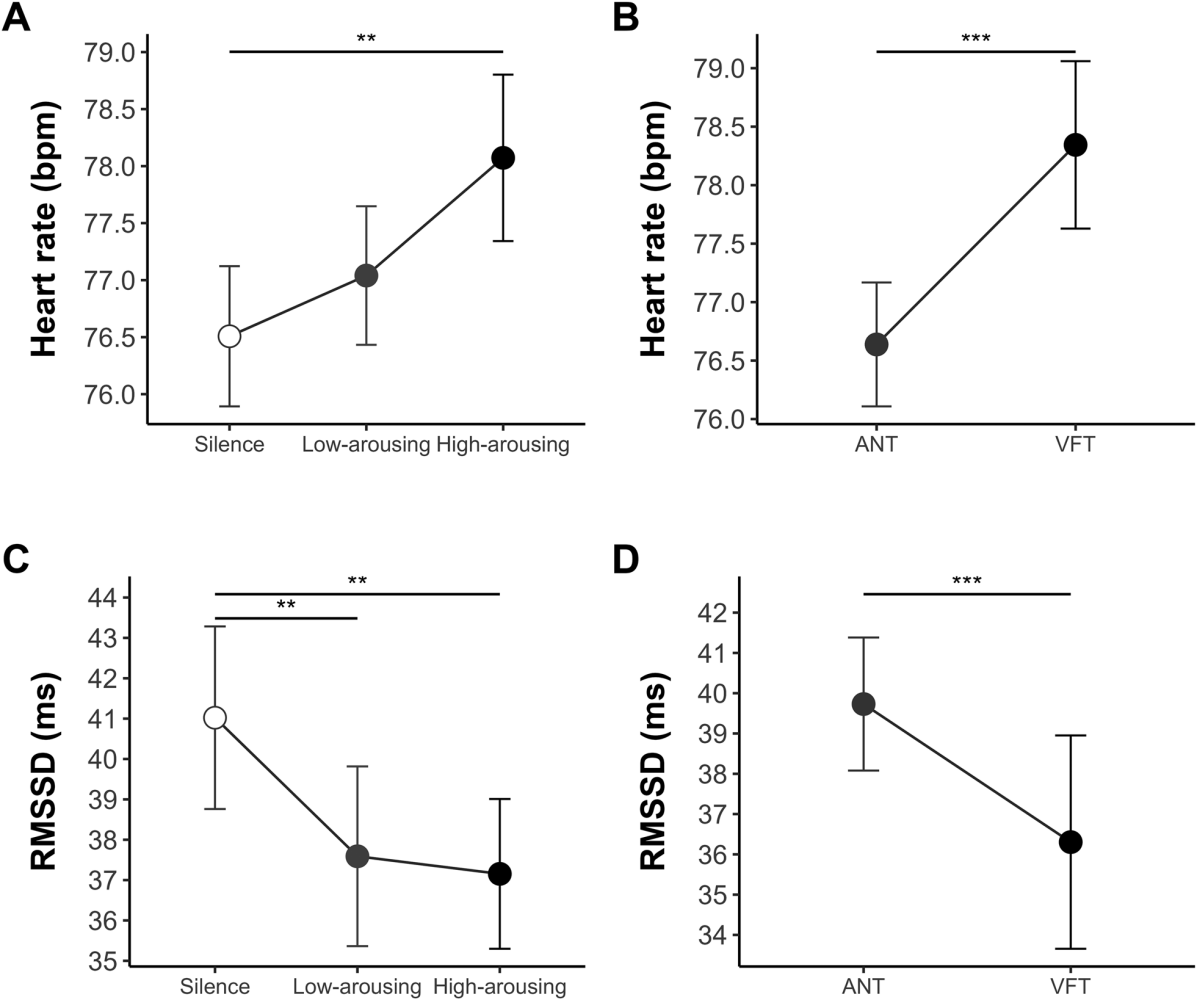
Hemodynamic activity indices as a function of sonic condition and task*. Note.* ANT = Attention Network Test; VFT = Verbal Fluency Task. Error bars indicate 95% confidence intervals. *p* < .05*, *p* < .01**, p ≤ .001***

The two-way rank-based RM ANOVA showed a significant effect of Sonic Condition on mean HR (*F*_*(2,66)*_ = 6.06, *p* = .004, *η*_*p*_^*2*^ = .16; see Fig. 3A), with a higher HR in the high-arousing condition (*M* = 78.06 bpm) compared to silence (*M* = 76.44 bpm; Wilcoxon’s* Z*_*(68)*_ = 600, *p*_adj_ = .001, *r* = .43). No significant differences emerged between the high- and low-arousing conditions (*M* = 76.97 bpm; Wilcoxon’s *Z*_*(68)*_ = 869, *p*_adj_ = .127, *r* = .23), nor between the low-arousing and silence conditions (Wilcoxon’s* Z*_*(68)*_ = 942, *p*_adj_ = .159, *r* = .17). Results showed also a significant effect of Task (*F*_*(1,33)*_ = 18.72, *p* < .001, *η*_*p*_^*2*^ = .36), with a lower HR in the ANT (*M* = 76.64 bpm) than in the VFT (*M* = 78.19 bpm, see Fig. 3B). The Task × Sonic Condition interaction was non-significant (*F*_*(2,66)*_ = 0.47, *p* = .630, *η*_*p*_^*2*^ = .01).

##### Heart rate variability

The 2-way rank-based RM ANOVA conducted on RMSSD showed a significant effect of Sonic Condition (*F*_*(2,66)*_ = 7.27, *p* = .001, *η*_*p*_^*2*^ = .18; see Fig. 3C), with a lower RMSSD during both the high- (*M* = 36.89 ms; Wilcoxon’s* Z*_*(68)*_ = 1737, *p*_adj_ = .001, *r* = .42) and low-arousing conditions (*M* = 36.86 ms; Wilcoxon’s* Z*_*(68)*_ = 1786, *p*_adj_ = .001, *r* = .45) compared to silence (*M* = 40.78 ms). No significant differences emerged between the high- and low-arousing conditions (Wilcoxon’s *Z*_*(68)*_ = 1199, *p*_adj_ = .876, *r* = .02). Results showed also a significant effect of Task (*F*_*(1,33)*_ = 23.49, *p* < .001, *η*_*p*_^*2*^ = .42), with a higher RMSSD in the ANT (*M* = 39.73 ms) than in the VFT (*M* = 35.07 ms, see Fig. 3D). The Task × Sonic Condition interaction was non-significant (*F*_*(2,66)*_ = 0.26, *p* = .773, *η*_*p*_^*2*^ = .01).

#### Respiratory activity

##### Respiratory rate

**Fig. 4:**
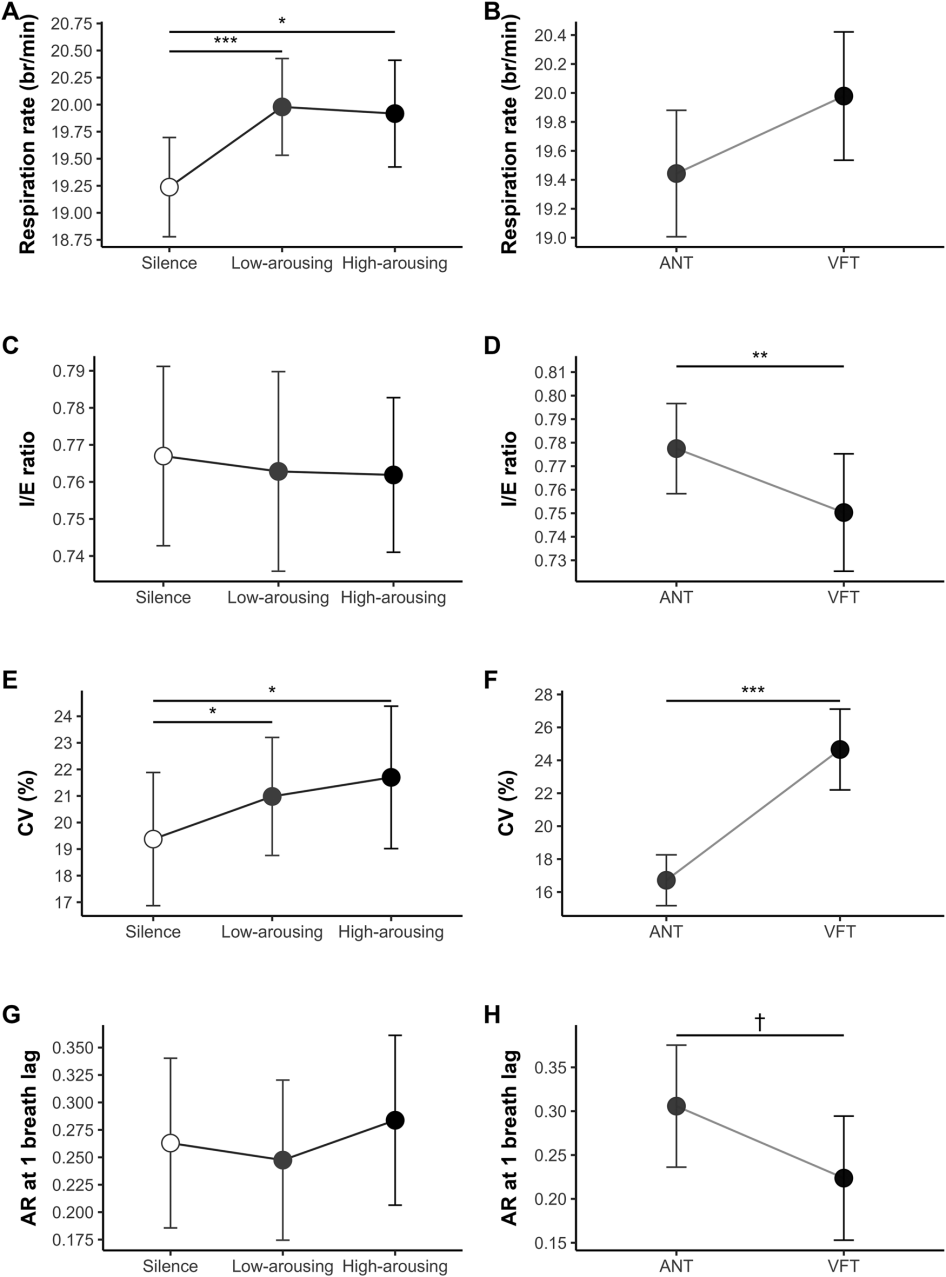
Respiratory activity indices as a function of sonic condition and task. *Note.* ANT = Attention Network Test; VFT = Verbal Fluency Task. Error bars indicate 95% confidence intervals. *p* < .10†, *p* < .05*, *p* < .01**, p ≤ .001***

Two-way rank-based RM ANOVA showed a significant effect of Sonic Condition on mean RR (*F*_*(2,66)*_ = 5.86, *p* = .005, *η*_*p*_^*2*^ = .13; see Fig. 4A), which was faster during both the high-arousing (*M* = 19.81 br/min; Wilcoxon’s* Z*_*(68)*_ = 744, *p*_adj_ = .018, *r* = .32) and low-arousing conditions (*M* = 19.95 br/min; Wilcoxon’s* Z*_*(68)*_ = 558, *p*_adj_ = .001, *r* = .46) compared to silence (*M* = 19.13 br/min). Results showed non-significant effects of Task (*F*_*(1,33)*_ = 1.89, *p* = .179, *η*_*p*_^*2*^ = .05) and Task × Sonic Condition interaction (*F*_*(2,66)*_ = 1.25, *p* = .293, *η*_*p*_^*2*^ = .04).

##### Inspiration/ Expiration ratio

The 2-way RM ANOVA conducted on I/E ratio showed a non-significant effect of Sonic Condition (*F*_*(2,66)*_ = 0.42, *p* = .660, *η*_*p*_^*2*^ = .01), but a significant effect of Task (*F*_*(1,33)*_ = 4.61, *p* = .039, *η*_*p*_^*2*^ = .12), with a higher I/E ratio during the ANT (*M* = 0.78) compared to the VFT (*M* = 0.74*,* see Fig. 4D). The Task × Sonic Condition interaction was non-significant (*F*_*(2,66)*_ = 0.95, *p* = .392, *η*_*p*_^*2*^ = .03).

##### Respiratory variability

The 2-way RM ANOVA conducted on CV showed a significant effect of Sonic Condition (*F*_*(2,66)*_ = 4.25, *p* = .018, *η*_*p*_^*2*^ = .11; see Fig. 4E), with a higher CV during the high- (*M* = 21.51;* t*_*(67)*_ = 2.85, *p*_adj_ = .018, *d* = 0.35) and low-arousing conditions (*M* = 20.52;* t*_*(67)*_ = 2.30, *p*_adj_ = .049, *d* = 0.28) compared to silence (*M* = 18.47). No significant differences emerged between the high- and low-arousing conditions (*M* = 20.52; *t*_*(67)*_ = 0.80, *p*_adj_ = .427, *d* = 0.10). Results revealed also a significant effect of Task (*F*_*(1,33)*_ = 43.98, *p* < .001, *η*_*p*_^*2*^ = .57; see Fig. 4F), with a lower CV in the ANT (*M* = 0.17) than in the VFT (*M* = 0.24). The Task × Sonic Condition interaction was non-significant (*F*_*(2,66)*_ = 1.88, *p* = .161, *η*_*p*_^*2*^ = .05).

The 2-way RM ANOVA performed on AR showed a marginally significant effect of Task (*F*_*(1,33)*_ = 3.30, *p* = .078, *η*_*p*_^*2*^ = .09) and non-significant effects of Sonic Condition (*F*_*(2,66)*_ = 0.24, *p* = .785, *η*_*p*_^*2*^ < .01) and Task × Sonic Condition interaction (*F*_*(2,66)*_ = 0.33, *p* = .717, *η*_*p*_^*2*^ = .01).

To summarize, the presence of background music induced higher physiological activation (lower heart rate variability, faster breathing rates and lower total respiratory variability) compared to silence. The presence of the high-arousing music induced the fastest heart rate. However, the increase in respiratory total variability was not accompanied by a change in autocorrelated variability, as previously observed in case of high cognitive load. There was also a significant effect of task. The observed pattern (faster heart rate, lower heart rate variability, increased total respiratory variability accompanied by a marginally significant decrease in AR) suggested an increase in cognitive load in the Verbal Fluency Task when contrasted to the attention network test. There was no interaction between sonic conditions and task nature.

### Behavioral measures

#### Attention Network Test (ANT)

Descriptive statistics and all other effects not directly related to our hypotheses are reported in Supplementary Tables S4, S5 and S6.

##### Network scores

**Fig. 5:**
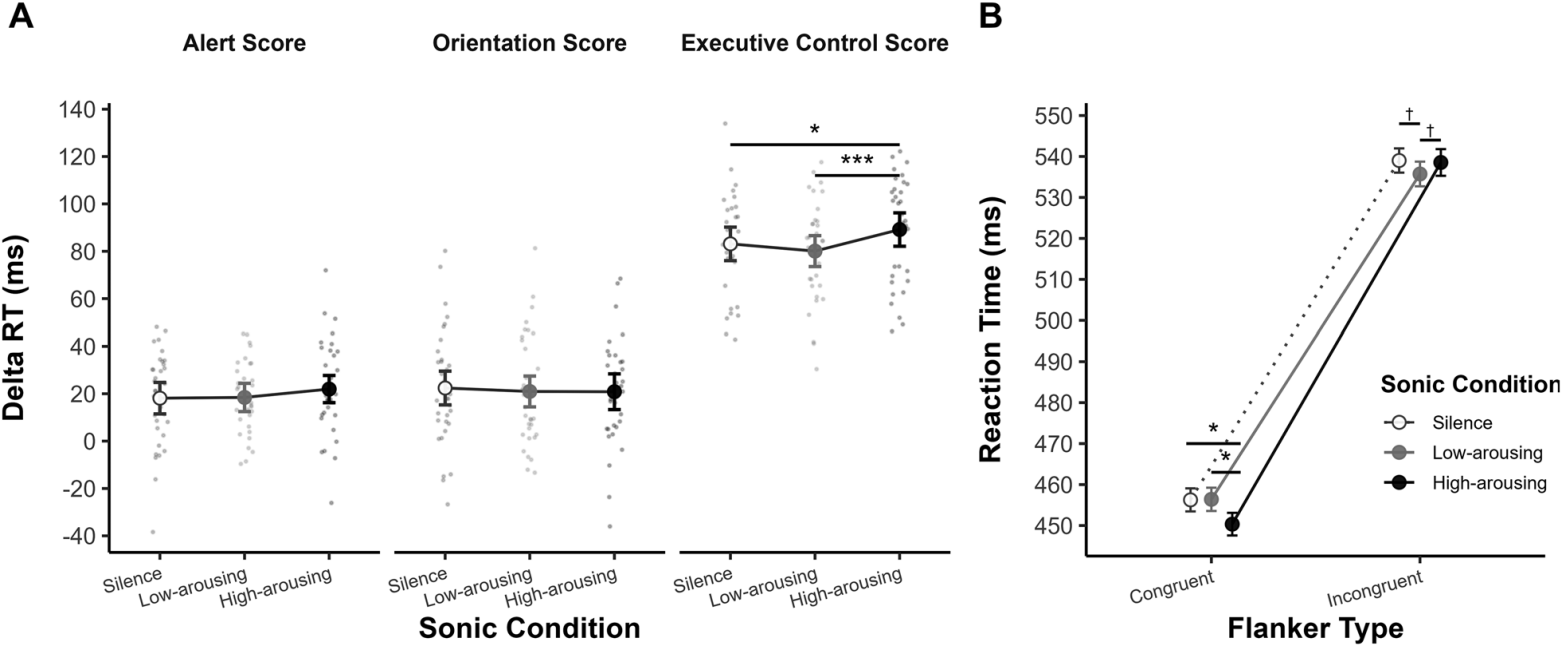
Network scores and reaction times as a function of sonic condition in the ANT. *Note.* Error bars indicate 95% confidence intervals. *p* < .10†, *p* < .05*, *p* < .01**, p ≤ .001***

The three network scores (alert, orientation, executive control) were subjected to separate 1-way RM ANOVAs (Sonic Condition [silence, low arousing, high arousing]). Statistical analyses revealed a significant effect of Sonic Condition on the executive control score (*F*_*(2,66)*_ = 6.78, *p* = .002, *η*_*p*_^*2*^ = .17), which was higher in the high-arousing sonic condition (*M* = 89.17 ms) than in the low-arousing (*M* = 80.07 ms; *t*_*(33)*_ = 4.03, *p*_adj_ < .001, *d* = .69) and silence conditions (*M* = 83.13 ms; *t*_*(33)*_ = 2.40, *p*_adj_ = .044, *d* = .41; see Fig. 5A). No significant differences emerged between the low-arousing and silence conditions (*t*_*(33)*_ = − 1.11, *p*_*adj*_ = .275, *d* = − .19). No significant effects of Sonic Condition were found on the alert (*F*_*(2,66)*_ = .62, *p* = .543, *η*_*p*_^*2*^ = .02) and orientation scores (*F*_*(2,66)*_ = .48, *p* = .622, *η*_*p*_^*2*^ = .01).

##### Reaction time

Reaction times were subjected to 3-way RM ANOVAs (Sonic Condition [silence, low arousing, high arousing] × Cue Type [no-cue, central, double, spatial] × Flanker Type [congruent, incongruent]). Results showed a non-significant effect of Sonic Condition on reaction times (*F*_*(2,66)*_ = .53, *p* = .589, *η*_*p*_^*2*^ = .02), but a significant Flanker × Sonic Condition interaction (*F*_*(2,66)*_ = 6.46, *p* = .003, *η*_*p*_^*2*^ = .16). Specifically, reaction times to congruent trials were faster in the high-arousing condition (*M* = 450.58 ms) compared to both the low-arousing (*M* = 456.77 ms; *t*_*(135)*_ = − 2.91, *p*_adj_ = .013, *d* = − .25) and silence conditions (*M* = 456.48 ms; *t*_*(135)*_ = 2.46, *p*_adj_ = .030, *d* = − .21; see Fig. 5B). No significant differences emerged between the low-arousing and silence conditions (*t*_*(135)*_ = 0.13, *p*_adj_ = .900, *d* = .01). Concerning the incongruent trials, results showed either marginally significant or non-significant differences among the three sonic conditions (high arousing vs. low arousing: *t*_*(135)*_ = 1.19, *p*_adj_ = .071, *d* = .10; high arousing vs. silence: *t*_*(135)*_ = 0.01, *p*_adj_ = .989, *d* < .01; low arousing vs. silence: *t*_*(135)*_ = − 1.17, *p*_adj_ = .071, *d* = − .10). As a whole, these results confirm the effects of high-arousing background music on the executive control scores.

##### Accuracy

Accuracy was subjected to a 3-way rank-based RM ANOVAs (Sonic Condition [silence, low arousing, high arousing] × Cue Type [no-cue, central, double, spatial] × Flanker Type [congruent, incongruent]). Results revealed a non-significant effect of Sonic Condition (*F*_*(2,66)*_ = .17, *p* = .847, *η*_*p*_^*2*^ = .01), suggesting no major differences in performance accuracy among silence (92.78%), low-arousing (92.82%) and high-arousing (91.76%) conditions. The interactions Sonic Condition × Cue Type (*F*_*(6,198)*_ = .53, *p* = .786, *η*_*p*_^*2*^ = .02) and Sonic Condition × Flanker Type (*F*_*(2,66)*_ = 1.41, *p* = .251, *η*_*p*_^*2*^ = .04) were also non-significant, suggesting that the sonic condition did not modulate the cue and flanker effects on accuracy.

#### Verbal Fluency Task (VFT)

##### Number of correct words

To analyze the effect of sonic conditions on mental flexibility and language access (VFT), the number of correct words produced was subjected to a 1-way RM ANOVA (Sonic Condition [silence, low arousing, high arousing]). Statistical analyses revealed a non-significant effect of Sonic Condition on the percentage of correct words produced (Friedman one-way RM ANOVA: *χ*^*2*^_*(2)*_ = .47, *p* = .789, *W* < .01). Participants produced on average 21.41 correct words (99.21% of the total number of words; *SD* = 3.94) in the high-arousing condition, 20.62 correct words (98.84%, SD = 3.73) in the low-arousing condition and 20.88 (98.77%, SD = 3.32) in the silence condition.

##### Inter-word delay

**Fig. 6:**
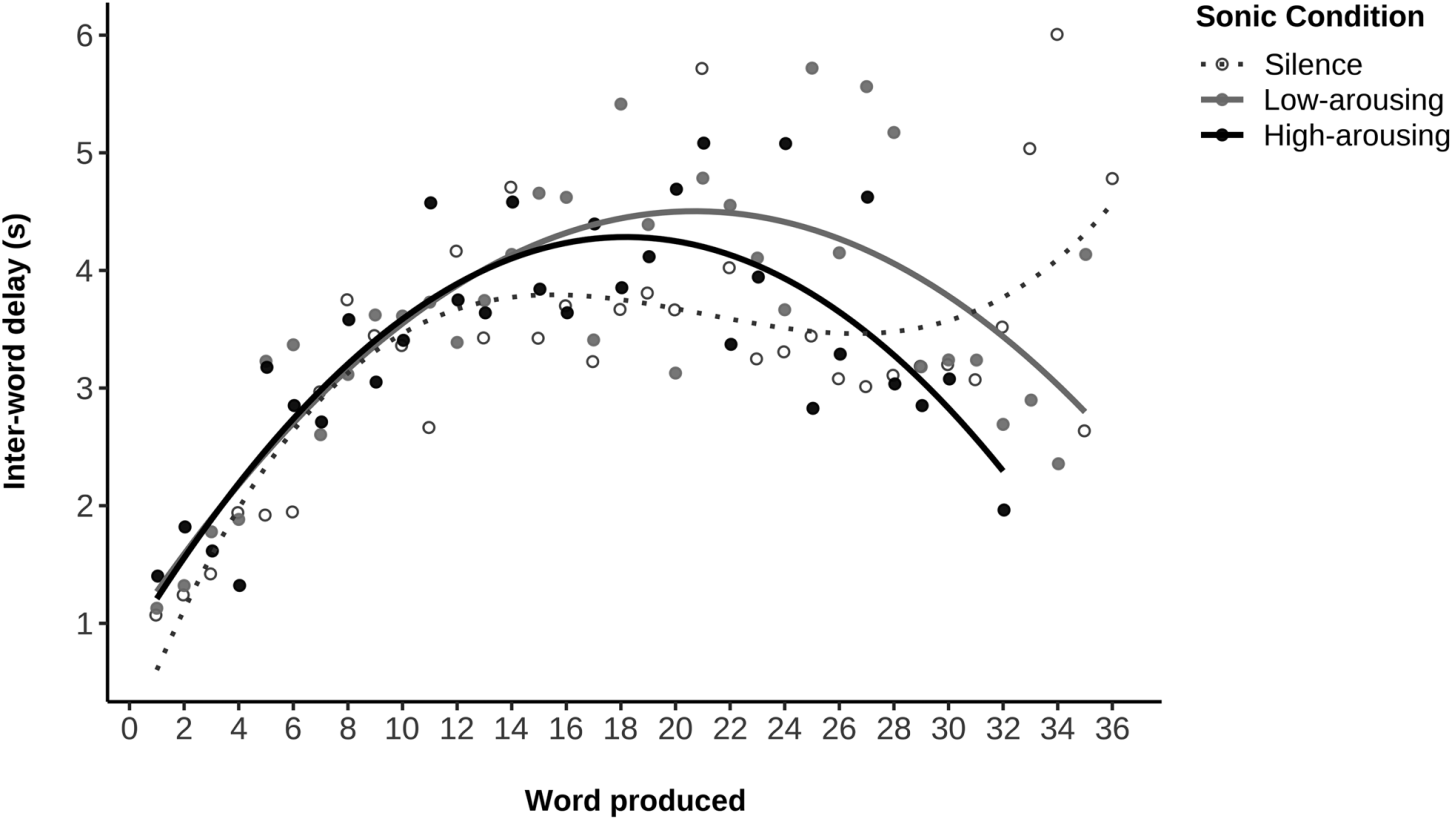
Inter-word delays in the VFT as a function of the sonic condition*. Note.* Low- and high-arousing conditions distributions were best fitted by a quadratic model, whereas a cubic model described best the distribution in the silence condition

The inter-word delay was analyzed by fitting linear, quadratic and cubic models. One data point was removed in the high-arousing condition as it impacted the quality of the fit. When analyzing the delay between each produced word, results showed that a quadratic model was the best fit for describing the distributions in the high- and low-arousing conditions (see Table 2; estimates are reported in Supplementary Table S7). As shown in Fig. 6, in both music conditions, the inter-word delay increased in the middle of the task and decreased at the end, suggesting that participants found words quickly at the beginning, more slowly in the middle, and then, slightly more quickly again toward the end of the task. On the contrary, the inter-word delay distribution in the silence condition was best fitted by a cubic model: the inter-word delay increased again at the end of the task, indicating a slowing down of word production fluidity.

**Table 2:** Model fitting results of inter-word delays as a function of sonic condition in the VFT

Effect	Sonic condition		
	Silence	Low arousing	High arousing
R^2^ Linear Model	0.32	0.17	0.14
R^2^ Quadratic Model	0.43	0.64	0.72
R^2^ Cubic Model	0.59	0.64	0.71
*Quadratic model*			
Linear effect	*F*_*(1,33)*_ = 18.66, *p* < *.*001***	*F*_*(1,32)*_ = 14.85, *p* < *.*001***	*F*_*(1,29)*_ = 14.11, *p* < *.*001***
Quadratic effect	*F*_*(1,33)*_ = 6.50, *p* = *.*016*	*F*_*(1,32)*_ = 41.59, *p* < *.*001***	*F*_*(1,29)*_ = 55.96, *p* < *.*001***
*Cubic model*			
Linear effect	*F*_*(1,32)*_ = 24.83, *p* < *.*001***	*F*_*(1,31)*_ = 14.40, *p* < *.* 001***	*F*_*(1,28)*_ = 13.70, *p* < *.*001***
Quadratic effect	*F*_*(1,32)*_ = 8.65, *p* = *.*006**	*F*_*(1,31)*_ = 40.34, *p* < *.*001***	*F*_*(1,28)*_ = 54.34, < *.*001***
Cubic effect	*F*_*(1,32)*_ = 11.92, *p* = *.*002**	*F*_*(1,31)*_ = 0.04, *p* = .838	*F*_*(1,28)*_ = 0.19, *p* = *.*670

*Note.* Linear model analyses were not fully reported as they yielded very low-quality fits (see R^2^ coefficients). *p* < .05*, *p* < .01**, *p* ≤ .001***

### Self-reported measures

All self-reported measures were submitted to 2-way RM ANOVAs (Sonic Condition [silence, low arousing, high arousing] × Task [ANT, VFT]). Descriptive statistics are reported in Table 3.

**Table 3:** Mean (and standard deviation) scores at the self-reported measures

Variable of interest	ANT			VFT		
	Silence	Low arousing	High arousing	Silence	Low arousing	High arousing
*NASA-TLX*						
Global score	8.04 (1.63)	8.24 (1.68)	9.30 (1.91)	9.29 (1.35)	9.13 (1.83)	9.36 (1.85)
Mental load	10.09 (3.79)	10.56 (3.39)	12.18 (4.14)	13.71 (2.72)	12.82 (3.84)	14.18 (2.42)
Physical demands	3.03 (2.23)	3.00 (2.91)	3.41 (2.74)	3.21 (2.47)	3.32 (2.55)	3.32 (2.28)
Time pressure	9.44 (3.89)	9.68 (4.32)	11.59 (4.92)	10.00 (3.46)	9.91 (4.40)	10.32 (3.93)
Performance	9.85 (4.74)	10.21 (5.10)	10.15 (5.23)	10.15 (4.44)	10.09 (4.14)	10.29 (4.42)
Effort	8.15 (3.86)	8.97 (3.79)	10.12 (4.06)	10.56 (3.12)	10.47 (3.03)	10.62 (3.53)
Frustration	7.71 (3.55)	7.03 (4.11)	8.38 (4.11)	8.15 (3.93)	8.15 (4.02)	7.44 (3.62)
*Subjective experience*						
Parasite thoughts	9.32 (5.45)	9.47 (5.04)	8.03 (5.29)	6.47 (4.61)	4.68 (3.44)	5.18 (3.55)
Task easiness	11.18 (4.56)	11.71 (4.09)	10.38 (3.89)	11.82 (3.78)	12.32 (2.89)	10.18 (2.99)
Task pleasantness	9.44 (5.73)	13.53 (5.48)	13.03 (4.07)	10.79 (4.50)	14.35 (4.24)	12.74 (3.28)

*Note:* ANT = Attention Network Test; VFT = Verbal Fluency Task

#### NASA-TLX

##### Global score

The two-way RM ANOVA showed a significant effect of Sonic Condition on the global score (*F*_*(2,66)*_ = 3.94, *p* = .024, *η*_*p*_^*2*^ = .11), which was higher in the high-arousing condition (*M* = 9.33) compared to the low-arousing (*M* = 8.68; *t*_*(67)*_ = 2.57, *p*_adj_ = .029, *d* = .31) and silence conditions (*M* = 8.67; *t*_*(67)*_ = 2.66, *p*_adj_ = .029, *d* = .32). No significant differences emerged between the low-arousing and silence conditions (*t*_*(67)*_ = 0.06, *p*_adj_ = .955, *d* = .01). Results revealed also a significant effect of Task (*F*_*(1,33)*_ = 4.37, *p* = .044, *η*_*p*_^*2*^ = .12), with a higher cognitive load score reported in the VFT (*M* = 9.26) than in the ANT (*M* = 8.53). The Sonic Condition × Task interaction was also significant (*F*_*(2,66)*_ = 3.60, *p* = .033, *η*_*p*_^*2*^ = .01). Specifically, participants reported a higher global cognitive load in the high-arousing condition than both the low-arousing (*t*_*(33)*_ = 2.99, *p*_adj_ = .010, *d* = .51) and silence conditions (*t*_*(33)*_ = 3.50, *p*_adj_ = .004, *d* = .60), but only in the ANT. No differences were found between the low-arousing condition and silence (*t*_*(33)*_ = 0.53, *p*_adj_ = .603, *d* = .09). No significant differences emerged among sonic conditions during the VFT (high vs. low arousing: *t*_*(33)*_ = 0.67, *p*_adj_ = .100, *d* = .12; high arousing vs. silence: *t*_*(33)*_ = 0.22, *p*_adj_ = .100, *d* = .04; low arousing vs. silence: *t*_*(33)*_ = − 0.46, *p*_adj_ = .100, *d* = − .08).

##### Items scores

**Fig. 7:**
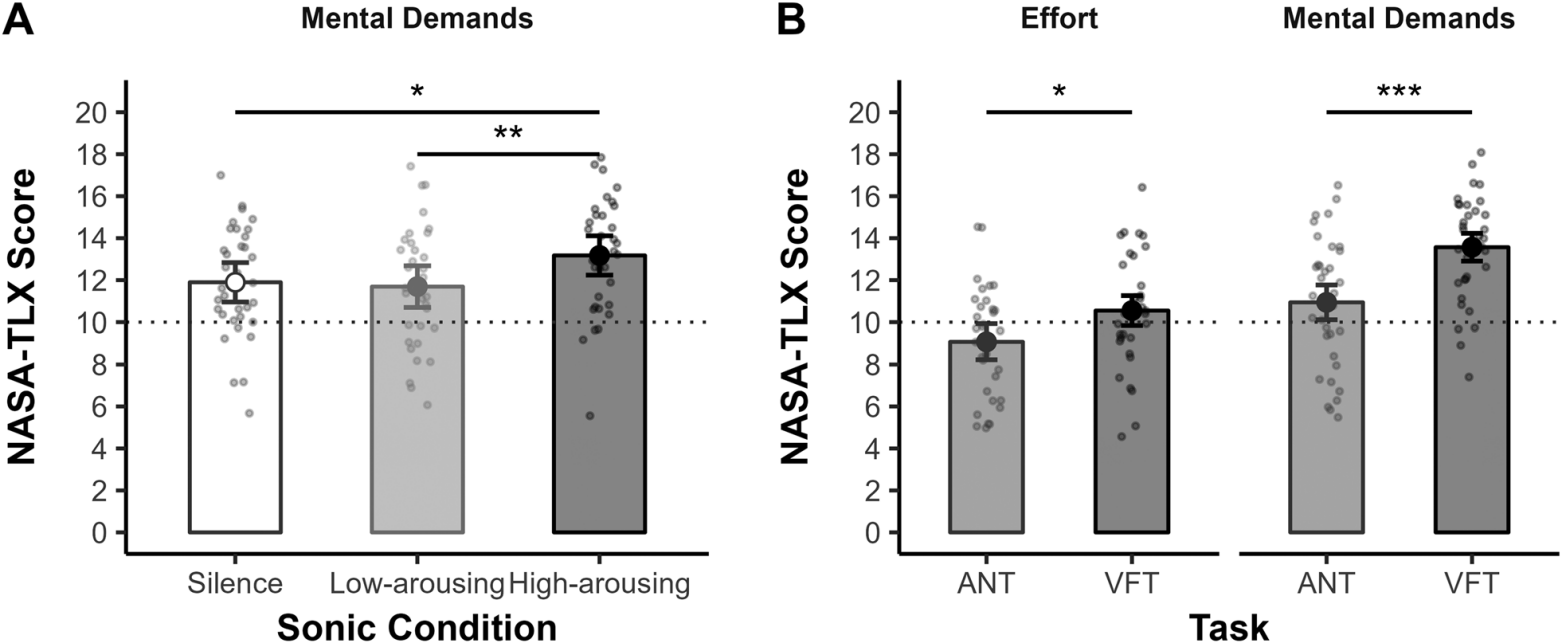
Effects of sonic condition and task at the NASA-TLX scale. *Note.* Error bars indicate 95% confidence intervals. Small, jittered points represent individual scores. The dotted horizontal line indicates the middle of the scale. *p* < .05*, *p* < .01**, *p* ≤ .001***

When looking at single-item scores, results suggested that the higher cognitive load experienced in the high-arousing condition was mainly due to greater mental demands. Indeed, results showed a significant effect of Sonic Condition (rank-based RM ANOVA: *F*_*(2,66)*_ = 5.29, *p* = .001, *η*_*p*_^*2*^ = .14; see Fig. 7A), with participants perceiving the tasks as more mentally demanding when performing them with the high-arousing excerpt (*M* = 13.18) than the low-arousing one (*M* = 11.69; Wilcoxon’s* Z*_*(68)*_ = 446, *p*_*adj*_ = .004, *r* = .39) or in silence (*M* = 11.90; Wilcoxon’s* Z*_*(68)*_ = 529.5, *p*_adj_ = .022, *r* = .29). No differences were found between the two latter conditions (Wilcoxon’s *Z*_*(68)*_ = 809, *p*_adj_ = .746, *r* = .05). Results revealed also an effect of Task, the participants perceiving the VFT (*M* = 13.57) as more mentally demanding than the ANT (*M* = 10.94; *F*_*(1,33)*_ = 17.19, *p* < .001, *η*_*p*_^*2*^ = .34; see Fig. 7B). The Sonic Condition × Task interaction was non-significant (*F*_*(2,66)*_ = 1.96, *p* = .150, *η*_*p*_^*2*^ = .06). In addition to greater mental demands, participants experienced also greater effort during the VFT (*M* = 10.55) compared to the ANT (*M* = 9.08; *F*_*(1,33)*_ = 4.49, *p* = .042, *η*_*p*_^*2*^ = .12; see Fig. 7B). The effects of Sonic Condition and the Sonic Condition × Task interaction on perceived effort were both non-significant (*F*_*(2,66)*_ = 1.12, *p* = .333, *η*_*p*_^*2*^ = .03 and *F*_*(1.6,52.2)*_ = 1.63, *p* = .209, *η*_*p*_^*2*^ = .05, respectively). No significant effects were found on any of the other items (see Supplementary Table S8).

#### Subjective experience during task execution

##### Parasite thoughts

**Fig. 8:**
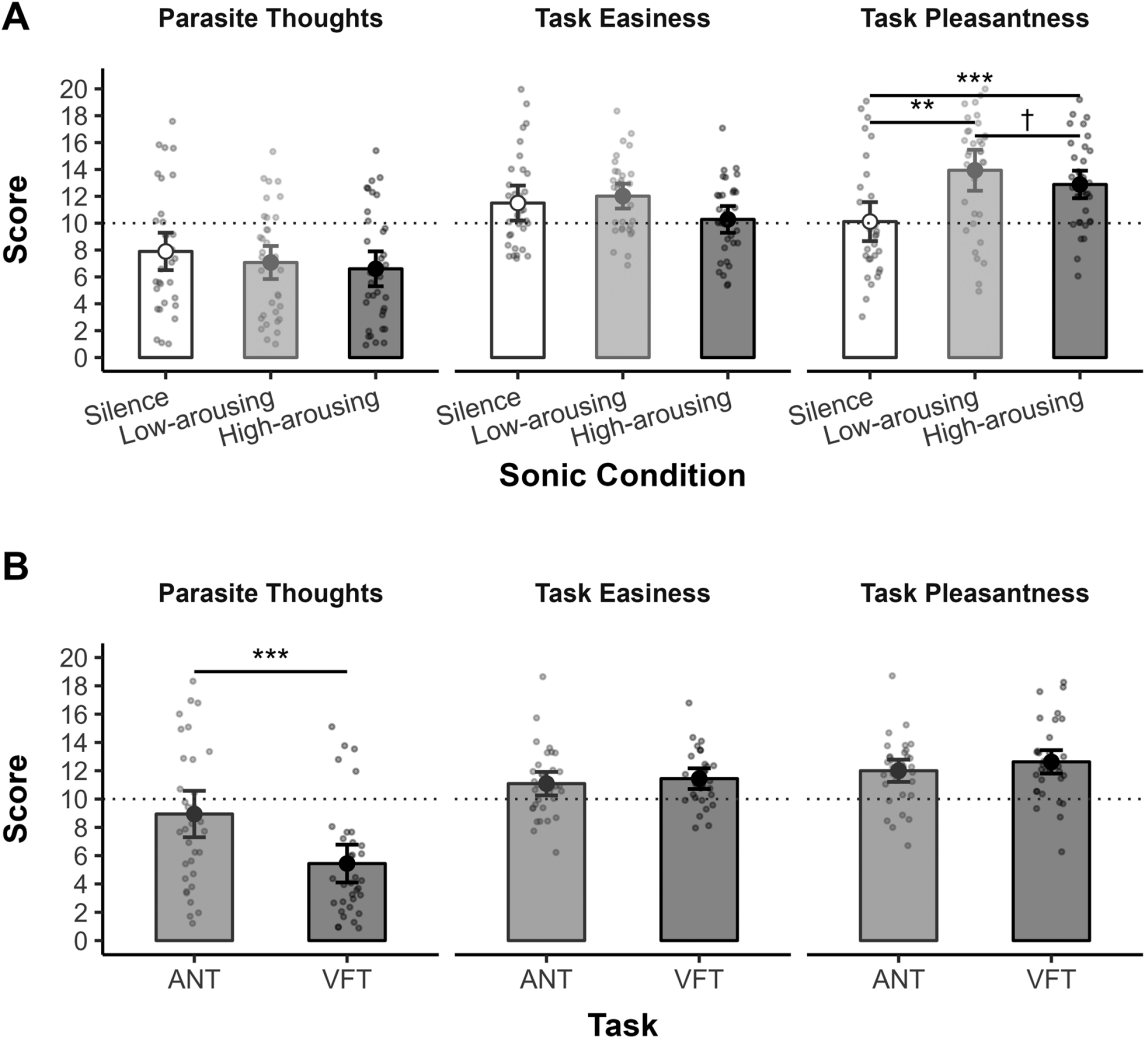
Effects of sonic condition and task on parasite thoughts occurrence, task peasantness and task easiness*. Note.* ANT = Attention Network Test; VFT = Verbal Fluency Task*.* Error bars indicate 95% confidence intervals. Small, jittered points represent individual scores. The dotted horizontal line indicates the middle of the scale. High scores indicate higher task pleasantness, easiness, and greater occurrence of parasite thoughts. *p* < .10†, *p* < .05*, *p* < .01**, p ≤ .001***

Results showed no significant effects of Sonic Condition on the occurrence of parasite thoughts (rank-based RM ANOVA: *F*_*(2,66)*_ = 2.23, *p* = .125, *η*_*p*_^*2*^ = .06). They showed a significant effect of Task (*F*_*(1,33)*_ = 14.92, *p* < .001, *η*_*p*_^*2*^ = .31), the participants experiencing more parasite thoughts in the ANT (*M* = 8.94) than the VFT (*M* = 5.44, see Fig. 8B). This effect was not modulated by the sonic condition, as the Sonic Condition × Task interaction was not significant (*F*_*(2,66)*_ = 2.16, *p* = .123, *η*_*p*_^*2*^ = .06).

##### Task easiness

Results showed no significant effects of the Task (*F*_*(1,33)*_ = 0.70, *p* = .409, *η*_*p*_^*2*^ = .02) and the Sonic Condition × Task interaction (*F*_*(2,66)*_ = 0.46, *p* = .634, *η*_*p*_^*2*^ = .01). The effect of the Sonic Condition (rank-based RM ANOVA: *F*_*(2,66)*_ = 2.67, *p* = .087, *η*_*p*_^*2*^ = .07) failed to reach significance, providing no evidence of a significant impact of the sonic environment on the perception of task easiness.

##### Task pleasantness

Results revealed an effect of Sonic Condition on task pleasantness (rank-based RM ANOVA: *F*_*(2,66)*_ = 9.09, *p* < .001, *η*_*p*_^*2*^ = .22), the participants rating the tasks as more pleasant to perform in both the high-arousing (*M* = 12.88; *Z*_*(68)*_ = 471, *p*_*adj*_ = .004, *r* = .36) and low-arousing conditions (*M* = 13.94; *Z*_*(68)*_ = 465, *p*_adj_ < .001, *r* = .50) compared to silence (*M* = 10.12, see Fig. 8A). The difference between the high- and low-arousing conditions was marginally significant (*Z*_*(68)*_ = 1130.5, *p*_adj_ = .064, *r* = .21). The effects of Task and Sonic Condition × Task interaction were both non-significant (*F*_*(1,33)*_ = 2.49, *p* = .124, *η*_*p*_^*2*^ = .07 and *F*_*(2,66)*_ = 1.03, *p* = .364, *η*_*p*_^*2*^ = .03 respectively).

### Exploratory analyses

In order to explore what mechanisms underlie the observed findings, we conducted exploratory multiple regression analysis. Based on the hypothesis that background music’s sonic energy increases mental demands but also available resources through its arousing and pleasure-providing potentials, we examined how these variables predicted changes in physiological activation. For each physiological index, we run regression models including sonic condition, task, mental demands and task pleasantness as predictors. These variables were selected as they showed significant effects on physiological activation.

The model run on respiratory CV was the only one to reach statistical significance (*F*_*(5,186)*_ = 10.22, *p* < .001, *R*^*2*^_multiple_ = .22 *R*^*2*^_adjusted_ = .19). Specifically, compared to the baseline, both the low-arousing (estimate = 3.83, SE = 1.58, *t* = 2.43, *p* = .016) and high-arousing sonic conditions (estimate = 4.56, SE = 1.56, *t* = 2.92, *p* = .004) significantly increased CV. Performing the VFT task was also associated with a significant increase in CV (estimate = 8.15, SE = 1.29, *t* = 6.32, *p* < .001). In contrast, greater mental demands (estimate = − 0.30, SE = 0.13, *t* = − 2.24, *p* = .026) and higher task pleasantness (estimate = − 0.46, SE = 0.14, *t* = − 3.32, *p* = .001) were associated with reductions in CV. However, all other models showed poor quality fit, as indicated by low R2, and failed to reach significance. Related results are reported in Supplementary Table S9.

It is worth noting that these analyses were exploratory and do not allow to draw robust conclusions. Future studies should address these questions using a more appropriate paradigm, larger sample size and explore interaction and mediation effects among predictors. Finally, musical expertise and gender effects on behavioral outcomes were also explored. Since the analyses did not reveal major modulations of these factors on the observed effects, results are reported in Supplementary Analyses 2 and 3.

## Discussion

This study assessed the impact of background music’s sonic energy (i.e., the arousing potential) on cognitive performances, perceived effort and task execution experience. The novelty was to operationalize music’s arousing potential by combining the analysis of arousal-related musical features and subjective ratings. The demands of the cognitive task were taken into account, contrasting attention to verbal fluency. Behavioral, physiological and self-reported measures were collected to obtain more comprehensive insights. Overall, findings suggested that when presented during a task, background music does not only have a distracting effect, but if correctly dosed in terms of sonic energy, it can mobilize resources, provide enjoyment and improve performances without detrimental cognitive load. Results are discussed in more detail in the following sections.

### Effects of sonic energy on physiological activation

Based on the distraction-conflict, arousal-and-mood and hedonic approaches (Gonzalez & Aiello, 2019; Husain et al., 2002; Scott et al., 2024), we expected background music to be not only resources consuming, but also resources providing, and even more if high arousing. Results partially confirmed this hypothesis, showing increased physiological activation in the presence of both the low- and high-arousing musical excerpts. Such increase was driven by higher heart rate variability, faster respiration rate and greater total respiration variability compared to silence. This autonomic pattern has been previously observed in situations of heightened attention or effort (e.g., alerted or awake states and REM sleep stages; Duschek et al., 2009; Gutierrez et al., 2016; Rostig et al., 2005) and was interpreted as an increase in metabolic needs, aiming at maintaining optimal levels of oxygen and carbon dioxide in response to external demands. However, despite a descriptive trend, inferential statistics did not show significant differences between high- and low-arousing excerpts, the latter being expected to induce a calmer state (Kirk et al., 2022). Such lack of a modulation effect by music sonic energy can be explained by the characteristics of our excerpts which, contrarily to other studies, were specifically designed for cognitive tasks (no lyrics, no seducing details, stable rhythmicity). As a consequence, the excerpt was high arousing but not enough to induce major autonomic changes.

A further question is whether such physiological activation reflects a negative stress response to distraction (as posited by the distraction-conflict approach; Gonzalez & Aiello, 2019) or a mobilization of available resources (Husain et al., 2002; Vigl et al., 2023). Addressing this question requires to integrate physiological, behavioral and self-reported data. First, no decrease in autocorrelated respiratory variability was found, contradicting previous observations in stressful and cognitively demanding situations (Grassmann, et al., 2016a, 2016b; Vlemincx et al., 2011). Second, no performance deterioration was observed with any musical excerpt in either task. Moreover, participants experienced greater pleasure in the presence of background music, with only a slight increase in mental demands limited to the attentional task. Finally, exploratory regressions suggested that while music sonic energy and task complexity increased respiratory variability (by heightening external demands), higher mental demands and pleasure decreased it, indicating the instauration of a sustained attention state (Grassmann, et al., 2016a, 2016b; Vlemincx et al., 2011). Taken together, these findings suggest that background music does systematically act as a stressor. Rather, it can enhance autonomic activity through an adaptative metabolic response to a richer informational context and affective reactions to music’s hedonic potential, which modulates arousal through enjoyment (Chabris, 1999; Husain et al., 2002; Lim & Park, 2018; Salimpoor et al., 2009). Nevertheless, further researches are needed to precisely disentangle distraction and resources mobilization effects, by identifying potential specific physiological patterns.

By collecting cardiac and respiratory measures, our work provided a direct quantification of background music effects on arousal levels, which are often assessed indirectly through questionnaires (Chee et al., 2024). Future studies should complete the present findings using more precise metabolic measurements (e.g., volumetric measures of ventilatory activity, Grassmann, et al., 2016a, 2016b) and tasks compatible with eye-tracking data collection to quantify cognitive load (Einhäuser, 2017; Kahneman, 1973; Mahanama et al., 2022). In our case, the frequent gaze shifts between the keyboard and the screen in the Verbal Fluency Task made the ocular signals unsuitable for robust and detailed analyses. Finally, individual differences in personality traits (e.g., extraversion/introversion, anxiety) should be considered, as they may influence physiological activation levels at rest and sensitivity to background music (e.g., Cassidy & Macdonald, 2007; Furnham & Strbac, 2002; Küssner, 2017; Van Diest et al., 2006).

### Effects of sonic energy on task performances

Our second hypothesis was that by mobilizing resources, background music should improve performances, task easiness and task pleasantness with only a minor increase in cognitive load. Results globally verified this hypothesis. The presence of both low- and high-arousing background music increased the pleasure experienced during task execution compared to a silent environment. The high-arousing background music elicited also a mild increase in perceived cognitive load compared to the two other conditions. Such effect was mainly observed in the ANT. Despite that, high-arousing music improved attentional performances, speeding up executive control processes with no accuracy loss. In the VFT, the presence of both the low- and high-arousing excerpt was associated with improved word retrieval performances across time. Interestingly, during this task cognitive load was not affected by the presence of background music. Such effect might be explained by the shorter duration of the Verbal Fluency Task (2 vs. 10 min in the attentional task), which might also represent a confounding factor explaining the lack of interaction effects on other measured variables. Overall and in line with previous studies (e.g., Gonzalez & Aiello, 2019), these outcomes suggest that energetic background music may consume a greater part of the available resources, requiring to manage a stronger attentional conflict. However, since performances were improved instead of deteriorated in both tasks, we can conclude that the sonic energy chosen in the present study was high enough to mobilize available resources without being deleterious. In support of this explanation, no changes in perceived task easiness emerged across sonic conditions.

### Effects of sonic energy as a function of task demands

Our third aim was to examine whether sonic energy effects depended on task demands. As hypothesized, the Attention Network Test (requiring attentional and decision-making processes) was rated as less demanding and induced lower physiological activation than the Verbal Fluency Task (requiring mental flexibility word retrieval processes). Coherently, participants reported experiencing more parasite thoughts in the attentional than the Verbal Fluency Task, which reflects the engagement of less resources that become available for mind-wandering (Gonzalez & Aiello, 2019; Levinson et al., 2012).

In line with our hypothesis, performances at the attentional task benefitted more from the high-energy music, leading to better executive control abilities. This improvement was driven by faster reaction times to congruent trials, with no deterioration of incongruent trials processing nor of performance precision (speed-accuracy trade-off). These results corroborate prior positive outcomes observed with fast and joyful musical excerpts on similar tasks (Fernandez et al., 2019; Marti-Marca et al., 2020). However, since the improvements were observed in the higher-level executive control abilities but not in the lower-level alert component, the present findings challenge the assumption that energetic music would benefit only tasks involving simple cognitive processes (e.g., Kiss & Linnell, 2023). They suggest that music’s effects are not limited to changes in alert state, which relies on the norepinephrinergic system and frontal, parietal and thalamic areas (de Souza Almeida et al., 2021; Fan et al., 2009; Fernandez et al., 2019), but may expand to more complex processes involving conflict-resolution and decision-making abilities, which rely on the dopaminergic system, dorsal anterior cingulate cortex (ACC) and lateral prefrontal cortices activation (de Souza Almeida et al., 2021; Fan et al., 2009; MacLeod et al., 2010). Nonetheless, these interpretations remain speculative and need to be precisely tackled in future neuroscientific studies.

Regarding the Verbal Fluency Task, the results provided partial support to our initial hypothesis. While we expected the low-energy excerpt to be the most suited for improving performances, results showed that both sonic energy levels impacted verbal fluency processes. First, and in contrast with previous similar studies (Cho, 2015; Ransdell & Gilroy, 2001), background music did not hinder the number of words produced. A potential explanation lies in the characteristics of excerpts used in these earlier studies, such as the musical structure complexity (e.g., vocal excerpts and instrumental adaptations in Ransdell & Gilroy, 2001) or the music’s potential to evoke contexts unrelated to work situations (e.g., *Gangnam Style* in Cho, 2015). To minimize distraction, we used musical excerpts specifically composed for cognitive tasks, avoiding lyrics, privileging a repetitive simple structure and controlling for a series of musical and acoustic features.

With such musical setting, the present study showed for the first time a beneficial impact of background music on the dynamics of verbal production processes. Specifically, by analyzing the time taken to reflect between each word produced, we observed that at the end of the task, word production fluency improved in the presence of both excerpts compared to silence, suggesting that music helped participants re-engage in the task. This improvement may be attributed to the enjoyment provided by music, which could have mobilized available resources, as well as to the music’s power to evoke emotions, which are in turn known to enhance memory (Bottiroli et al., 2014; Jäncke, 2008). Nonetheless, these interpretations remain speculative and additional research—using longer writing tasks and analyzing semantic content patterns in responses—could provide shed light on the mechanisms underlying these promising results.

### Constraints on generality

Since this study was conducted on a French population, results can be generalized to European and Western musical cultures, but less to populations having non-Western musical culture. The sample used in the present study is representative of a young studying or working population (between 18 and 40 years old), more susceptible to listening to music during their daily cognitive activities (Goltz & Sadakata, 2021; Kotsopoulou & Hallam, 2010). Generalization to younger or older populations should also be approached more cautiously due to the neurocognitive specificities of each group, such as the automatization level of core abilities (e.g., comprehension, memorization) in younger students (< 18 years old) or aging-related impairments in executive function control and distractions inhibition in older populations (> 60 years old; Fraser & Bherer, 2013). Despite several encouraging findings in these populations (de la Mora Velasco et al., 2023; Fernandez et al., 2019), more studies are needed to define the interaction between their neurocognitive specificities and background music characteristics. Finally, the present study employed two controlled musical excerpts and two specific cognitive tasks. Diversifying the musical excerpts and using tasks tackling other core cognitive functions would significantly expand the present findings.

## Conclusions

Our study suggests that the sonic energy (or arousing potential) of background music can positively influence cognitive task performances. When optimally dosed and tailored to task nature, music’ sonic energy mobilizes available resources, enhances pleasure and positively impacts cognitive performances, competing only minimally with the ongoing task. Furthermore, by improving task execution conditions, background music could make some tasks less burdensome, reduce procrastination and boost intrinsic motivation, encouraging individuals to engage in task for the enjoyment their execution provides. Our research extends previous findings by collecting multidimensional measures, by objectively quantifying music’s arousing potential (through subjective ratings and musical-acoustic analysis) and physiological responses. Our outcomes offer a broader view of the interplay between background music’s distraction effects and its hedonic and mood-related benefits. They also pave the way for future holistic investigations of background music effects on cognitive performances, which should consider personality traits, attentional or learning disorders, longer-lasting and ecologic tasks. The present findings find applications in various music-related domains, including music therapy, psycho-acoustic and music use in educational contexts.

## Supplementary Information

Additional file 1.

## Data Availability

Data and code are shared openly as part of the publication of the article. They are accessible at the following link: 10.5281/zenodo.13374906. This study’s design and related analyses were not pre-registered.

## Footnotes

1 **The ollen music sophistication index** (OMSI, by Ollen, 2006) is a validated 10-item scale used to assess musical sophistication, namely individuals’ musical background and expertise. Item 10 alone ("*Which option defines you the most?*", forced choice of 1 out of 6 options) proved to be sufficient to reliably estimate the degree of musical sophistication. It strongly predicts the scores at the full OMSI scale and the Gold-MSI scale (Müllensiefen et al., 2014), the other main reference scale for assessing musical sophistication and training (Zhang & Schubert, 2019).
