# Supplementary material for: The sonic energy of background music impacts cognitive performances: a behavioral and physiological investigation
Source: Cogn Res Princ Implic. 2025 Nov 18;10:80. doi: 10.1186/s41235-025-00676-9 (PMC12627320; doi:10.1186/s41235-025-00676-9)
Supplement: Supplementary file 1 — Additional file 1. [file 41235_2025_676_MOESM1_ESM.docx]

**Supplementary Material**

**The Sonic Energy of Background Music Impacts Cognitive Performances: A Behavioral and Physiological Investigation**

## Supplementary Analysis 1: Subjective and Musical Assessment of the Excerpts’ Sonic Energy

**Subjective Assessment.** The two musical excerpts were selected from a pre-validated database developed by Filboost^©^, along with 2 high- and 2 low-arousing alternative excerpts. The 9 musical excerpts were evaluated by 10 naïve participants, who listened to 40-second extracts capturing the main theme of each musical composition. Participants rated the excerpts in terms of perceived *arousing potential* (“How did you find the excerpt?”, using a 20-point scale ranging from 0-Very relaxing to 20-Very stimulating*)* and *valence* (“How did you find the excerpt?”, from 0-Very Negative to 20-Very Positive*)*. Following the results, we selected the two excerpts that diverged the most on the arousing potential continuum (high-arousing excerpt: *M* = 14.40, *SD* = 1.65; low-arousing excerpt: *M* = 4.10, *SD* = 1.73) and that had global positive valence (high-arousing excerpt: *M* = 14.10, *SD* = 2.08; low-arousing excerpt: *M* = 14.80, *SD* = 2.70). Arousing potential and valence levels of the two excerpts were rated by the participants of the main experiment as well.

**Musical Features Analysis**. In addition to the subjective evaluation, a musical features analysis was conducted using the Matlab MIRToolbox version 1.8 (Lartillot et al., 2008) to quantify the sonic energy of the two excerpts and know the musical “signature” of these excerpts for future researches. Based on previous works (Fernandez et al., 2019; Gomez & Danuser, 2007; Schubert, 1999; Trost et al., 2015), we selected 9 parameters relative to the rhythmic (tempo, pulse clarity), temporal (root mean square [RMS, or loudness], event density, zero-crossing rate) and spectral dimensions (brightness, roll-off, spectral centroid, spectral entropy). These parameters were selected as they were found to be more strongly correlated with the perceived arousing potential of musical excerpts. Some of these features (event density, pulse clarity, brightness, loudness) proved to be accurate in distinguishing tender from joyful musical excerpts, which correspond to positive low- and positive high-arousing dimensions respectively (Fernandez et al., 2019). Supplementary Table 1 provides a detailed description of each parameter, its interpretation and the MIR function computing it.

## Supplementary Table S1

*Description of the Musical Features Extracted and the MIR Functions Used*

| Parameter | Musical interpretation | | MIR function | Description |
| --- | --- | --- | --- | --- |
| Rhythmic Dimension |  |  | |  |
| *Tempo* | Speed at which the music is played | mirtempo with the parameter ‘metre’ (Lartillot & Grandjean, 2019) | | Number of beats per minute (bpm), estimated from periodicities in the event detection curve |
| *Pulse Clarity* | How clearly the beat or rhythm are detectable | mirpulseclarity | | Rhythmic clarity, estimated from the strength of the beats identified by the mirtempo function |
|  |  |  | |  |
| Temporal Dimension |  |  | |  |
| *Root Mean Square* | Energy of the sound, loudness | mirrms (with a normalization to maximum) | | Global energy of the signal, quantified by taking the root average of the square of the amplitude (or root-mean-square, RMS) |
| *Event Density* | Density of notes within a time unit | mireventdensity | | Average frequency of musical events (i.e., number of events detected per second) |
| *Zero Crossing Rate* | Index of the noisiness of the sound | mirzcr | | Number of times the signal crosses the X-axis (i.e., changes sign) |
|  |  |  | |  |
| Spectral Dimension |  |  | |  |
| *Brightness* | Sharpness of the sound | mirbrightness | | Amount of high frequencies (above 1500Hz - Juslin, 2000). |
| *Roll-off* | Another index of brightness | mirrolloff | | The frequency below which a certain percentage (85%, Tzanetakis & Cook, 2010) of the total spectral energy of the signal is concentrated |
| *Spectral Centroid* | Tendance of the sound to have more bass, medium or treble frequencies | mircentroid | | Measure of the "center of mass" of the spectrum of a signal, reflecting where the majority of the frequency content is concentrated |
| *Spectral Entropy* | Noisiness of the sound | mirentropy | | ‘Peakiness’ (or flatness) of the spectrum, evaluated based on the Shannon Theory |

*Note*. Audio files were extracted using the function ‘miraudio’ of the MIR Toolbox.

In line with prior studies (Alluri et al., 2012; Fernandez et al., 2019; Trost et al., 2015) long-term (tempo, pulse clarity and event density) and short-term features (rms, zero-crossing rate, spectral centroid, entropy, brightness and roll-off) were computed in two separate window analyses. Long-term features – unfolding over extended periods of time – were extracted using a 3.1s time-window (corresponding to the average duration of an ANT trial), considering an overlap between successive windows of 67% (corresponding to a 1s shifted window). Short-term parameters– changing rapidly, varying over short durations– were computed using a 50-ms time-window with an overlap of 50% (25-ms shifted window). The musical excerpts were cut into successive intervals of 30 seconds, for which mean values for each parameter were calculated. The two excerpts were then compared on each parameter by means of unpaired Wilcoxon rank-sum tests. Supplementary Table 2 below shows mean parameters values (averaged across all the 30s intervals) and statistical results, confirming that the two excerpts differed significantly in all arousal-related parameters.

## Supplementary Table S2

*Mean (SD) Values and Statistical Results for each Parameter as a Function of the Excerpt*

| Parameter | Excerpt | |  | Unpaired Wilcoxon Rank-Sum Test | | |
| --- | --- | --- | --- | --- | --- | --- |
|  | Low-Arousing | High-Arousing |  | *Z* | *p* | *r* |
| Rhythmic Dimension | | | | | |  |
| Tempo | 60 (0.80) | 120 (0.03) |  | 0 | <.001*** | 0.86 |
| Pulse Clarity | 0.27 (0.02) | 0.70 (0.10) |  | 0 | <.001*** | 0.86 |
|  |  |  |  |  |  |  |
| Temporal Dimension | | | | | |  |
| Root Mean Square | 0.16 (0.01) | 0.20 (0.04) |  | 71 | <.001*** | 0.59 |
| Event Density | 1.82 (0.19) | 2.89 (0.62) |  | 0 | <.001*** | 0.86 |
| Zero Crossing Rate | 385.73 (88.74) | 630.08 (390.57) |  | 87 | <.001*** | 0.53 |
|  |  |  |  |  |  |  |
| Spectral Dimension |  | |  |  |  |  |
| Brightness | 0.19 (0.02) | 0.29 (0.07) |  | 2 | <.001*** | 0.85 |
| Roll off | 2346 (272) | 4480 (1116) |  | 1 | <.001*** | 0.85 |
| Spectral Centroid | 1381 (90) | 2163 (543) |  | 1 | <.001*** | 0.85 |
| Spectral Entropy | 0.63 (0.01) | 0.69 (0.04) |  | 10 | <.001*** | 0.82 |

*Note*. *r* = Wilcoxon effect size (< 0.3: small effect, < 0.5: moderate effect, ≥ 0.5: large effect). *p* < .05 *, *p* < .01**, *p* ≤ .001***.

## Supplementary Table S3

*Mean (and Standard Deviations) of Physiological Indices as a Function of Sonic Condition and Task*

| Index | ANT | | |  | VFT | | |
| --- | --- | --- | --- | --- | --- | --- | --- |
|  | Silence | Low-arousing | High-arousing |  | Silence | Low-arousing | High-arousing |
| Hemodynamic activity | | | | | | | |
| HR (bpm) | 75.85 (2.66) | 76.49 (2.69) | 77.58 (3.27) |  | 77.62 (2.51) | 77.94 (2.62) | 79.02 (3.22) |
| RMSSD (ms) | 42.20 (8.42) | 38.81 (11.09) | 38.19 (7.30) |  | 37.95 (11.98) | 32.95 (6.40) | 34.30 (7.91) |
|  |  |  |  |  |  |  |  |
| Respiratory activity | | | | | | | |
| RR (br/min) | 18.78 (1.52) | 19.80 (1.42) | 19.75 (1.58) |  | 19.48 (1.25) | 20.10 (1.60) | 19.88 (1.68) |
| I/E ratio | 0.77 (0.08) | 0.78 (0.07) | 0.77 (0.05) |  | 0.75 (0.09) | 0.73 (0.08) | 0.74 (0.07) |
| CV (%) | 15.29 (3.98) | 17.94 (5.35) | 16.91 (6.41) |  | 21.65 (5.95) | 23.09 (6.81) | 26.12 (9.27) |
| AR | 0.32 (0.28) | 0.29 (0.24) | 0.31 (0.30) |  | 0.21 (0.27) | 0.20 (0.28) | 0.26 (0.26) |

*Note*. ANT= Attention Network Test; VFT= Verbal Fluency Task; HR = Heart Rate; RMSSD = Root Mean Square of Successive Differences between normal heartbeats; RR = Respiratory Rate; I/E = Ratio of Inspiration to Expiration times; CV = Coefficient of Variation; AR = Autocorrelated variability. CV and AR are calculated with respect to RR.

## Supplementary Table S4

*Mean (Standard Deviation) Results at the ANT as a Function of Sonic Condition*

| Variable of Interest | Sonic Condition | | |
| --- | --- | --- | --- |
|  | Silence | Low-Arousing | High-Arousing |
|  |  |  |  |
| *Network Scores (ms)* | | | |
| Network |  |  |  |
| Alert | 18.13 (19.06) | 18.44 (17.10) | 21.97 (16.48) |
| Orientation | 22.41 (20.44) | 20.96 (18.67) | 20.85 (21.63) |
| Executive control | 83.13 (20.29) | 80.07 (18.73) | 89.17 (20.20) |
|  |  |  |  |
| *Reaction Time (ms)* | | | |
| Cue Type |  |  |  |
| No cue | 513.49 (70.91) | 509.69 (72.28) | 509.49 (75.88) |
| Central | 494.93 (74.27) | 494.75 (73.28) | 490.80 (79.06) |
| Double | 494.90 (77.77) | 491.55 (75.57) | 487.61 (77.31) |
| Spatial | 472.21 (71.47) | 473.49 (73.08) | 469.00 (73.30) |
| Flanker Type |  |  |  |
| Congruent | 456.27 (65.94) | 456.40 (66.65) | 450.34 (64.75) |
| Incongruent | 539.01 (63.13) | 535.73 (63.87) | 538.53 (67.71) |
| Global Reaction Time | 493.78 (88.17) | 492.18 (87.66) | 489.17 (91.27) |
|  |  |  |  |
| *Accuracy (%)* | | | |
| Cue Type |  |  |  |
| No cue | 93.84 (6.58) | 93.27 (6.12) | 92.90 (6.62) |
| Central | 91.72 (5.86) | 92.19 (6.00) | 90.98 (6.72) |
| Double | 92.19 (5.87) | 91.63 (5.83) | 91.07 (6.79) |
| Spatial | 93.38 (6.69) | 94.20 (4.69) | 92.09 (7.16) |
| Flanker Type |  |  |  |
| Congruent | 98.71 (5.74) | 98.90 (5.40) | 98.85 (4.35) |
| Incongruent | 86.85 (6.52) | 86.74 (7.65) | 84.67 (9.05) |
| Global Accuracy | 92.78 (3.60) | 92.82 (2.99) | 91.76 (3.72) |

## Supplementary Table S5

*Reaction Times: Results of Three-Way Repeated-Measures ANOVA and Pairwise Comparisons in the Attention Network Test*

| Effect | Statistic | *p* | *p_adj_* | Effect size |
| --- | --- | --- | --- | --- |
| Omnibus 3-way ANOVA |  |  |  |  |
| Flanker Type | *F_(1,33)_* = 627.20 | <.001*** | - | *η_p_^2^* = .95 |
| Cue Type | *F_(1.8,58.6)_* = 67.30 | <.001*** | - | *η_p_^2^* = .67 |
| Sonic Condition | *F_(2,66)_* = 0.53 | .589 | - | *η_p_^2^* = .02 |
| Flanker Type × Cue Type | *F_(3,99)_* = 10.30 | <.001*** | - | *η_p_^2^* = .24 |
| Flanker Type × Sonic Condition | *F_(2,66)_* = 6.46 | .003** | - | *η_p_^2^* = .16 |
| Cue Type × Sonic Condition | *F_(6,198)_* = 0.66 | .680 | - | *η_p_^2^* = .02 |
| Flanker Type × Cue Type × Sonic Condition | *F_(6,198)_* = 0.82 | .554 | - | *η_p_^2^* = .02 |
|  |  |  |  |  |
| Simple Effect of Flanker Type |  |  |  |  |
| Congruent vs. Incongruent | *t_(407)_* = -59.38 | <.001*** | - | *d* = -2.94 |
|  |  |  |  |  |
| Simple Effect of Cue Type |  |  |  |  |
| Central vs. Double | *t_(204)_* = 1.29 | .200 | .200 | *d* = .90 |
| Central vs. No Cue | *t_(204)_* = -9.61 | <.001 | <.001*** | *d* = -.67 |
| Central vs. Spatial | *t_(204)_* = 11.91 | <.001 | <.001*** | *d* = .83 |
| Double vs. No Cue | *t_(204)_* = -11.12 | <.001 | <.001*** | *d* = -.78 |
| Double vs. Spatial | *t_(204)_* = 12.23 | <.001 | <.001*** | *d* = .86 |
| No Cue vs. Spatial | *t_(204)_* = 18.84 | <.001 | <.001*** | *d* = 1.32 |
|  |  |  |  |  |
| Flanker Type × Cue Type |  |  |  |  |
| Congruent Flanker |  |  |  |  |
| Central vs. Double | *t_(101)_* = 0.96 | 0.341 | 0.341 | *d* = .10 |
| Central vs. No Cue | *t_(101)_* = -8.86 | <.001 | <.001*** | *d* = -.88 |
| Central vs. Spatial | *t_(101)_* = 6.92 | <.001 | <.001*** | *d* = .69 |
| Double vs. No Cue | *t_(101)_* = -10.82 | <.001 | <.001*** | *d* = -1.07 |
| Double vs. Spatial | *t_(101)_* = 6.34 | <.001 | <.001*** | *d* = .69 |
| No Cue vs. Spatial | *t_(101)_* = 12.93 | <.001 | <.001*** | *d* = 1.28 |
| Incongruent Flanker |  |  |  |  |
| Central vs. Double | *t_(101)_* = 0.87 | 0.389 | 0.389 | *d* = .09 |
| Central vs. No Cue | *t_(101)_* = -4.92 | <.001 | <.001*** | *d* = -.49 |
| Central vs. Spatial | *t_(101)_* = 10.12 | <.001 | <.001*** | *d* = 1.00 |
| Double vs. No Cue | *t_(101)_* = -5.56 | <.001 | <.001*** | *d* = -.55 |
| Double vs. Spatial | *t_(101)_* = 11.45 | <.001 | <.001*** | *d* = 1.13 |
| No Cue vs. Spatial | *t_(101)_* = 13.68 | <.001 | <.001*** | *d* = 1.35 |

*Note.* Reaction times were analyzed through parametric tests. *t* = Student’s *t*; *p_adj_* = Holm-Bonferroni adjusted p-values; *d* = Cohen’s *d* (with 0.2 = small effect, 0.5 = moderate effect, 0.8 = large effect). *p* < .05 *, *p* < .01**, *p* ≤ .001***.

## Supplementary Table S6

*Accuracy: Results of Three-Way Repeated-Measures ANOVA and Pairwise Comparisons in the Attention Network Test*

| Effect | Statistic | *p* | *p_adj_* | Effect size |
| --- | --- | --- | --- | --- |
| Omnibus 3-way ANOVA |  |  |  |  |
| Flanker Type | *F_(3,99)_* = 3.75 | .013* | - | *η_p_^2^* = .10 |
| Cue Type | *F_(1,33)_* = 121.30 | <.001*** | - | *η_p_^2^* = .79 |
| Sonic Condition | *F_(2,66)_* = 0.17 | .847 | - | *η_p_^2^* = .01 |
| Flanker Type × Cue Type | *F_(3,99)_* = 8.29 | <.001*** | - | *η_p_^2^* = .20 |
| Flanker Type × Sonic Condition | *F_(6,198)_* = 0.53 | .786 | - | *η_p_^2^* = .02 |
| Cue Type × Sonic Condition | *F_(2,66)_* = 1.41 | .251 | - | *η_p_^2^* = .04 |
| Flanker Type × Cue Type × Sonic Condition | *F_(6,198)_* = 0.79 | .575 | - | *η_p_^2^* = .02 |
|  |  |  |  |  |
| Simple Effect of Flanker Type |  |  |  |  |
| Congruent vs. Incongruent | *Z_(407)_* = 55561 | <.001*** | - | *r* = .80 |
|  |  |  |  |  |
| Simple Effect of Cue Type |  |  |  |  |
| Central vs. Double | *Z_(203)_* = 2747.5 | .910 | 1.00 | *r* = *.05* |
| Central vs. No Cue | *Z_(203)_* = 2091.0 | .003 | .015* | *r* = *.21* |
| Central vs. Spatial | *Z_(203)_* = 1669.5 | .003 | .015* | *r* = *.18* |
| Double vs. No Cue | *Z_(203)_* = 2281.5 | .013 | .039* | *r* = *.18* |
| Double vs. Spatial | *Z_(203)_* = 1594.5 | .003 | .015* | *r* = *.20* |
| No Cue vs. Spatial | *Z_(203)_* = 2722.5 | .981 | 1.00 | *r* = *.02* |
|  |  |  |  |  |
| Flanker Type × Cue Type |  |  |  |  |
| Congruent Flanker |  |  |  |  |
| Central vs. Double | *Z_(101)_* = 119.0 | .074 | .368 | *r* = .21 |
| Central vs. No Cue | *Z_(101)_* = 334.5 | .986 | 1.00 | *r* < .01 |
| Central vs. Spatial | *Z_(101)_* = 117.0 | .186 | .568 | *r* = .17 |
| Double vs. No Cue | *Z_(101)_* = 246.5 | .053 | .319 | *r* = .20 |
| Double vs. Spatial | *Z_(101)_* = 75.5 | .703 | 1.00 | *r* = .05 |
| No Cue vs. Spatial | *Z_(101)_* = 131.5 | .142 | .568 | *r* = .17 |
| Incongruent Flanker |  |  |  |  |
| Central vs. Double | *Z_(101)_* = 1657.0 | .559 | 1.00 | *r* = .04 |
| Central vs. No Cue | *Z_(101)_* = 812.5 | .002 | .007** | *r* = .33 |
| Central vs. Spatial | *Z_(101)_* = 899.0 | .005 | .014* | *r* = .23 |
| Double vs. No Cue | *Z_(101)_* = 1095.0 | .001 | .006** | *r* = .35 |
| Double vs. Spatial | *Z_(101)_* = 1003.0 | .001 | .006** | *r* = .33 |
| No Cue vs. Spatial | *Z_(101)_* = 1622.5 | .531 | 1.00 | *r* = .06 |

*Note.* Accuracy was analyzed using non-parametric tests. *Z* = Wilcoxon paired signed-rank test’ *Z*; *p_adj_* = Holm-Bonferroni adjusted p-values; *r* = Wilcoxon effect size (with < 0.3: small effect, < 0.5: moderate effect, ≥ 0.5: large effect). *p* < .05 *, *p* < .01**, *p* ≤ .001***.

## Supplementary Table S7

*Coefficient Estimates of Quadratic and Cubic Models for Inter-Word Delays in the Verbal Fluency Task*

| Index | Sonic Condition | | | | | | | | | | |
| --- | --- | --- | --- | --- | --- | --- | --- | --- | --- | --- | --- |
|  | Silence | | |  | Low-arousing | | |  | High-arousing | | |
|  | Estimate | *t* | *p* |  | Estimate | *t* | *p* |  | Estimate | *t* | *p* |
| Quadratic Model | |  |  |  |  |  |  |  |  |  |  |
| b0 | 1.36 | 3.02 | .005** |  | 0.93 | 2.49 | .018* |  | 0.84 | 2.58 | .015* |
| b1 | 0.20 | 3.52 | .001 |  | 0.35 | 7.19 | <.001*** |  | 0.38 | 8.17 | <.001*** |
| b2 | -0.004 | -2.55 | .016 |  | -0.01 | -6.45 | <.001*** |  | -0.01 | -7.48 | <.001*** |
|  |  |  |  |  |  |  |  |  |  |  |  |
| Cubic Model |  |  |  |  |  |  |  |  |  |  |  |
| b0 | 0.03 | 0.06 | .95 |  | 0.85 | 1.60 | .120 |  | 0.98 | 2.11 | .044* |
| b1 | 0.60 | 4.75 | <.001*** |  | 0.37 | 2.91 | .007** |  | 0.33 | 2.73 | .011* |
| b2 | -0.03 | -3.88 | <.001*** |  | -0.01 | -1.23 | .228 |  | -0.01 | -0.79 | .434 |
| b3 | 0.0005 | 3.45 | .002** |  | 0.0002 | 0.21 | .838 |  | 0.0001 | -0.43 | .669 |

*Note.* *p* < .05 *, *p* < .01**, *p* ≤ .001***.

## Supplementary Table S8

*Results of Two-Way Repeated-Measures ANOVAs conducted on NASA-TLX Single-Item Scores*

| Index | Effect |  | Statistic | *p* | *η_p_^2^* |
| --- | --- | --- | --- | --- | --- |
|  |  |  |  |  |  |
| **Mental demands** | **Sonic Condition** |  | ***F_(2,66)_* = 5.86** | **.005**** | **0.15** |
|  | **Task** |  | ***F_(1,33)_* = 18.64** | **<.001***** | **0.36** |
|  | Sonic Condition × Task |  | *F_(2,66)_* = 1.77 | .179 | 0.05 |
|  |  |  |  |  |  |
| Physical demands | Sonic Condition |  | *F_(1.6,52.1)_* = 0.43 | .607 | 0.01 |
|  | Task |  | *F_(1,33)_* = 0.21 | .651 | 0.01 |
|  | Sonic Condition × Task |  | *F_(2,66)_* = 0.79 | .458 | 0.02 |
|  |  |  |  |  |  |
| Time pressure | Sonic Condition |  | *F_(2,66)_* = 2.43 | .096 | 0.07 |
|  | Task |  | *F_(1,33)_* = 0.04 | .839 | <0.01 |
|  | Sonic Condition × Task |  | *F_(2,66)_* = 1.53 | .225 | 0.04 |
|  |  |  |  |  |  |
| Performance | Sonic Condition |  | *F_(2,66)_* = 0.12 | .891 | <0.01 |
|  | Task |  | *F_(1,33)_* = 0.03 | .891 | <0.01 |
|  | Sonic Condition × Task |  | *F_(2,66)_* = 0.07 | .937 | <0.01 |
|  |  |  |  |  |  |
| **Effort** | Sonic Condition |  | *F_(2,66)_* = 1.29 | .281 | 0.04 |
|  | **Task** |  | ***F_(1,33)_* = 4.26** | **.047*** | **0.11** |
|  | Sonic Condition × Task |  | *F_(1.6,52.6)_* = 1.74 | .191 | 0.05 |
|  |  |  |  |  |  |
| Frustration | Sonic Condition |  | *F_(2,66)_* = 0.21 | .809 | 0.01 |
|  | Task |  | *F_(1,33)_* = 0.07 | .787 | <0.01 |
|  | Sonic Condition × Task |  | *F_(2.66)_* = 1.87 | .162 | 0.01 |

*Note*. *p* < .05 *, *p* < .01**, *p* ≤ .001***.

## Supplementary Table S9

*Exploratory Multiple Regression Analysis*

| Index and Effect of Interest | Estimate | Standard Error | *t* | *p* | Overall Model |
| --- | --- | --- | --- | --- | --- |
| HR |  |  |  |  |  |
| Intercept | 72.80 | 2.65 | 27.47 | <.001*** | *R²_multiple_* = 0.03 |
| Sonic Condition: Low-arousing | 0.03 | 1.73 | 0.02 | .984 | *R²_adjusted_* < 0.01 |
| Sonic Condition: High-arousing | 0.88 | 1.71 | 0.51 | .610 | *F_(5,186)_* = 1.03 |
| Task: VFT | 1.22 | 1.42 | 0.86 | .390 | *p* = .399 |
| Mental Demands | 0.18 | 0.15 | 1.23 | .219 |  |
| Task Pleasantness | 0.17 | 0.15 | 1.10 | .272 |  |
|  |  |  |  |  |  |
| RMMSD |  |  |  |  |  |
| Intercept | 52.27 | 5.52 | 9.48 | <.001*** | *R²_multiple_* = 0.04 |
| Sonic Condition: Low-arousing | -1.60 | 3.60 | -0.45 | .657 | *R²_adjusted_* = 0.01 |
| Sonic Condition: High-arousing | -1.93 | 3.56 | -0.54 | .589 | *F_(5,186)_* = 1.48 |
| Task: VFT | -1.44 | 2.95 | -0.49 | .625 | *p* = .200 |
| Mental Demands | -0.52 | 0.31 | -1.69 | .092 |  |
| Task Pleasantness | -0.49 | 0.31 | -1.55 | .123 |  |
|  |  |  |  |  |  |
| RR |  |  |  |  |  |
| Intercept | 20.27 | 0.87 | 23.36 | <.001*** | *R²_multiple_* = 0.03 |
| Sonic Condition: Low-arousing | 0.78 | 0.57 | 1.37 | .171 | *R²_adjusted_* < 0.01 |
| Sonic Condition: High-arousing | 0.96 | 0.56 | 1.72 | .088 | *F_(5,186)_* = 1.04 |
| Task: VFT | 0.63 | 0.46 | 1.35 | .178 | *p* = .397 |
| Mental Demands | -0.05 | 0.05 | -0.96 | .338 |  |
| Task Pleasantness | -0.05 | 0.05 | -1.09 | .277 |  |
|  |  |  |  |  |  |
| I/E ratio |  |  |  |  |  |
| Intercept | 0.82 | 0.04 | 22.05 | <.001*** | *R²_multiple_* = 0.02 |
| Sonic Condition: Low-arousing | 0.01 | 0.02 | 0.43 | .671 | *R²_adjusted_* < 0.01 |
| Sonic Condition: High-arousing | 0.004 | 0.02 | 0.17 | .864 | *F_(5,186)_* = 0.88 |
| Task: VFT | -0.02 | 0.02 | -0.97 | .333 | *p* = .494 |
| Mental Demands | -0.001 | 0.002 | -0.68 | .498 |  |
| Task Pleasantness | -0.003 | 0.002 | -1.51 | .132 |  |
|  |  |  |  |  |  |
| **CV** |  |  |  |  |  |
| **Intercept** | **22.08** | **2.41** | **9.15** | **<.001***** | ***R²_multiple_* = 0.22** |
| **Sonic Condition: Low-arousing** | **3.83** | **1.58** | **2.43** | **.016*** | ***R²_adjusted_*** = **0.19** |
| **Sonic Condition: High-arousing** | **4.56** | **1.56** | **2.92** | **.004**** | ***F_(5,186)_*** = **10.22** |
| **Task: VFT** | **8.15** | **1.29** | **6.32** | **<.001***** | ***p* < .001***** |
| **Mental Demands** | **-0.30** | **0.13** | **-2.24** | **.026*** |  |
| **Task Pleasantness** | **-0.46** | **0.14** | **-3.32** | **.001***** |  |
|  |  |  |  |  |  |
| AR |  |  |  |  |  |
| Intercept | 0.17 | 0.09 | 1.89 | .060 | *R²_multiple_* = 0.04 |
| Sonic Condition: Low-arousing | -0.05 | 0.06 | -0.91 | .365 | *R²_adjusted_* = 0.02 |
| Sonic Condition: High-arousing | -0.02 | 0.06 | -0.35 | .724 | *F_(5,186)_* = 1.60 |
| Task: VFT | -0.11 | 0.05 | -2.31 | .022* | *p* = .161 |
| Mental Demands | 0.01 | 0.01 | 1.62 | .108 |  |
| Task Pleasantness | 0.01 | 0.01 | 1.45 | .148 |  |

*Note:* Model formula: *Physiological Activation* *Index ~ Sonic Condition + Task + Mental Demands + Task Pleasantness.* HR = Heart Rate; RMSSD = Root Mean Square of Successive Differences between normal heartbeats; RR = Respiratory Rate; I/E = Ratio of Inspiration to Expiration times; CV = Coefficient of Variation; AR = Autocorrelated variability. CV and AR are calculated with respect to RR. *p* < .05 *, *p* < .01**, *p* ≤ .001***.

## Supplementary Analysis 2: Exploratory analysis of Musical Expertise Effects

Since musical expertise was reported to mediate the distraction effect of background music on cognitive performances (Patston & Tippett, 2011), we explored whether this factor interacted with the observed effects. Our sample was composed at 68% by non-musicians or music-loving non-musicians and at 32% by amateur musicians. No professional musicians participated in this study. Below are reported only the significant effects of musical expertise. All other effects are reported in Supplementary Table S8.

**Reaction times**. In the ANT, the RM ANOVA conducted on reaction times revealed a significant Musical Expertise × Sonic Condition interaction (*F_(2,64)_* = 4.48, *p* = .015, *η_p_^2^* = .12). Musicians’ reaction times were slower in the low- (*M* = 489 ms, SD = 63) compared to the silence (*M* = 482 ms, SD = 61; *t_(87)_* = 2.67, *p_adj_* = .018, *d* = 0.29) and high-arousing conditions (*M* = 476 ms, SD = 63; *t_(87)_* = 5.26, *p_adj_* < .001, *d* = 0.56), with a significant difference between the latter two (*t_(87)_* = 2.67, *p_adj_* = .018, *d* = 0.29). Conversely, non-musicians reacted faster in the low-arousing condition (*M_mus_* = 502 ms, SD = 60; *t_(183)_* = -2.85, *p_adj_* = .015, *d* = -0.21) than in silence (*M* = 507 ms, SD = 61; *t_(183)_* = -3.18, *p_adj_* = .002, *d* = -0.41). No significant differences emerged between the low- and high-arousing conditions (*M* = 505 ms, SD = 64; *t_(183)_* = -2.02, *p_adj_* = .089, *d* = -0.15), nor between the silence and high-arousing ones (*t_(183)_* = 0.75, *p_adj_* = .452, *d* = 0.06).

Results revealed also a significant Musical Expertise × Sonic Condition × Cue interaction (*F_(6,192)_* = 2.20, *p* = .044, *η_p_^2^* = .06). Specifically, in the presence of a central cue, musicians reacted slower in the low- (*M* = 500 ms, SD = 61) than the high-arousing (*M* = 481 ms, SD = 62; *t_(21)_* = 5.64, *p_adj_* < .001, *d* = 1.20) and silence conditions (*M* = 484 ms, SD = 57; *t_(21)_* = 3.40, *p_adj_* = .005, *d* = 0.73), with no significant difference between the latter two (*t_(21)_* = 0.67, *p_adj_* = .510, *d* = 0.14). In the presence of a spatial cue, musicians were slower in the low- (*M* = 468 ms, SD = 58) compared to the high- arousing condition (*M* = 447 ms, SD = 55; *t_(21)_* = 4.32, *p_adj_* = .001, *d* = 0.92). All other comparisons were non-significant.

**Accuracy**. The non-parametric RM ANOVA conducted on accuracy revealed a main effect of Musical Expertise (*F_(1.32)_* = 14.38, *p* <.001, *η_p_^2^* = .31), with musicians being overall less accurate (*M*= 88.43 %, SD = 14.49) than non-musicians (*M* = 94.37 %, SD = 8.11). The Musical Expertise × Sonic Condition interaction was also significant (*F_(2,64)_* = 5.45, *p* = .007, *η_p_^2^* = .15), with musicians performing less accurately in the high- (*M* = 86.55 %, SD = 15.97) than the low-arousing condition (*M* = 89.55 %, SD = 15.19; *Z* = 875, *p* = .025, *r* = .33). No significant differences emerged between the high-arousing and silence conditions (*M* = 89.20 %, SD = 13.36; *Z* = 1068, *p_adj_* = .051, *d* = 0.20), nor between the silence and low-arousing ones (*Z* = 456, *p_adj_* = .67, *d* = 0.08). All comparisons for non-musicians were non-significant (Silence vs. Low-arousing: *M_silence_* = 94.49 %, SD = 7.45, *M_low_* = 94.38 %, SD = 7.56; *Z* = 2239, *p_adj_* = 1.00, *d* = 0.02; Silence vs. High-arousing: *M_high_* = 94.25 %, SD = 9.24; Z = 1584, *p_adj_* = 1.000, *d* = 0.05; Low- vs. High-arousing: *M* = 89.20 %, SD = 13.36; Z = 1766, *p_adj_* = 1.000, *d* = .05).

**Inter-word delay**. In the VFT, results showed that musicians’ inter-word delays were better predicted by a cubic model in the silence and low-arousing conditions, but by a quadratic model in the high-arousing one, suggesting an improvement of verbal fluidity in this latter condition. Non-musicians presented the inverse pattern, namely a quadratic trend in the silence and low-arousing conditions and a cubic trend in the high-arousing one. Supplementary Table S9 reports *R*², coefficients and significance tests details.

Taken together, these results suggest that musicians and non-musicians seem differently impacted by background music. In the attention task, musicians are faster but less accurate in both silence and high-arousing conditions. In the ANT, results revealed significant effects of musical expertise on reaction times and accuracy, but not on network scores. In the verbal fluency task, they seem to benefit more from the high-arousing condition, while non-musicians benefit more from the low-arousing one. Crucially, no significant effects of musical expertise emerged when analyzing the ANT network scores, nor in any of the self-reported scales). These results suggest that musician may be more sensitive to the sonic energy of background music. However, these findings remain exploratory and need to be corroborated by more solid analyses conducted on a more balanced sample and including professional musicians.

## Supplementary Table S10

*Inferential Statistics for Each Dependent Variable, Including the Musical Expertise as Between-Subject Factor*

| Effect | Statistic | *p* | *η_p_^2^* |
| --- | --- | --- | --- |
| *Network Scores* |  |  |  |
| Alert |  |  |  |
| Musical Expertise | *F_(1,32)_* < 0.01 | .953 | < .01 |
| Sonic Condition | *F_(2,64)_* = 0.48 | .623 | .02 |
| Musical Expertise × Sonic Condition | *F_(2,64)_* = 0.02 | .983 | < .01 |
| Orientation |  |  |  |
| Musical Expertise | *F_(1,32)_* = 2.33 | .137 | .07 |
| Sonic Condition | *F_(2,64)_* = 0.04 | .965 | < .01 |
| Musical Expertise × Sonic Condition | *F_(2,64)_* = 0.34 | .717 | .01 |
| Executive Control |  |  |  |
| Musical Expertise | *F_(1,32)_* = 0.05 | .821 | < .01 |
| **Sonic Condition** | ***F_(2,64)_* = 6.64** | **.002**** | **.17** |
| Musical Expertise × Sonic Condition | *F_(2,64)_* = 0.28 | .756 | < .01 |
|  |  |  |  |
| *Reaction Times* |  |  |  |
| Musical Expertise | *F_(1,32)_* = 2.46 | .127 | .07 |
| **Flanker Type** | ***F_(1,32)_* = 530.29** | **<.001***** | **.94** |
| **Cue Type** | ***F_(1.8,57.2)_* = 63.94** | **<.001***** | **.67** |
| Sonic Condition | *F_(2,64)_* = 1.47 | .237 | .04 |
| Musical Expertise × Flanker Type | *F_(1,32)_* = 0.03 | .876 | < .01 |
| Musical Expertise × Cue Type | *F_(1.8,57.2)_* = 1.30 | .280 | .04 |
| **Musical Expertise × Sonic Condition** | ***F_(2,64)_* = 4.84** | **.015*** | **.12** |
| **Flanker Type × Cue Type** | ***F_(3,96)_* = 10.83** | **<.001***** | **.25** |
| **Flanker Type × Sonic Condition** | ***F_(2,64)_* = 6.40** | **.003**** | **.17** |
| Cue Type × Sonic Condition | *F_(6,192)_* = 1.44 | .203 | .04 |
| Musical Expertise × Flanker Type × Cue Type | *F_(3,96)_* = 1.51 | .217 | .05 |
| Musical Expertise × Flanker Type × Sonic Condition | *F_(2,64)_* = 0.28 | .758 | < .01 |
| **Musical Expertise × Cue Type × Sonic Condition** | ***F_(6,192)_* = 2.20** | **.044*** | **.06** |
| Flanker Type × Cue Type × Sonic Condition | *F_(6,192)_* = 1.01 | .420 | .03 |
| Musical Expertise × Flanker Type × Cue Type × Sonic Condition | *F_(6,192)_* = 1.71 | .120 | .05 |
|  |  |  |  |
| *Accuracy* |  |  |  |
| **Musical Expertise** | ***F_(1,32)_* = 14.38** | **<.001***** | **.31** |
| **Flanker Type** | ***F_(1,32)_* = 302.68** | **<.001***** | **.90** |
| **Cue Type** | ***F_(3,96)_* = 4.80** | **.004**** | **.13** |
| Sonic Condition | *F_(2,64)_* = 2.12 | .128 | .06 |
| **Musical Expertise × Flanker Type** | ***F_(1,32)_* = 6.74** | **.014*** | **.17** |
| **Musical Expertise × Cue Type** | ***F_(3,96)_* = 2.89** | **.039*** | **.08** |
| **Musical Expertise × Sonic Condition** | ***F_(2,64)_* = 5.45** | **.007**** | **.15** |
| **Flanker Type × Cue Type** | ***F_(3,96)_* = 6.09** | **<.001***** | **.16** |
| Flanker Type × Sonic Condition | *F_(2,64)_* = 0.60 | .553 | .02 |
| Cue Type × Sonic Condition | *F_(6,192)_* = 1.00 | .428 | .03 |
| Musical Expertise × Flanker Type × Cue Type | *F_(3,96)_* = 0.45 | .719 | .01 |
| Musical Expertise × Flanker Type × Sonic Condition | *F_(2,64)_* = 0.30 | .744 | < .01 |
| Musical Expertise × Cue Type × Sonic Condition | *F_(6,192)_* = 0.62 | .717 | .02 |
| Flanker Type × Cue Type × Sonic Condition | *F_(6,192)_* = 1.27 | .272 | .04 |
| Musical Expertise × Flanker Type × Cue Type × Sonic Condition | *F_(6,192)_* = 1.35 | .237 | .04 |
|  |  |  |  |
| *Percentage of Correct Words Produced* |  |  |  |
| Musical Expertise | *F_(1,32)_* = 0.31 | .580 | .01 |
| Sonic Condition | *F_(2,64)_* = 0.43 | .653 | .01 |
| Musical Expertise × Sonic Condition | *F_(2,64)_* = 0.61 | .547 | .02 |
|  |  |  |  |
| *NASA-TLX – Global Score* |  |  |  |
| Musical Expertise | *F_(1,32)_* = 0.00 | 1.000 | < .01 |
| **Sonic Condition** | ***F_(2,64)_* = 4.25** | **.019*** | **.12** |
| Task | *F_(1,32)_* = 2.66 | .113 | .08 |
| Musical Expertise × Sonic Condition | *F_(2,64)_* = 0.67 | .514 | .02 |
| Musical Expertise × Task | *F_(1,32)_* = 0.82 | .373 | .03 |
| Sonic Condition × Task | *F_(2,64)_* = 4.58 | .014* | .13 |
| Musical Expertise × Sonic Condition × Task | *F_(2,64)_* = 1.08 | .346 | .03 |
|  |  |  |  |
| *Subjective Experience* |  |  |  |
| Parasite Thoughts |  |  |  |
| Musical Expertise | *F_(1,32)_* < 0.01 | .978 | < .01 |
| Sonic Condition | *F_(1.7,53.7)_* = 1.67 | .201 | .05 |
| **Task** | ***F_(1,32)_* = 10.86** | **.002**** | **.25** |
| Musical Expertise × Sonic Condition | *F_(1.7,53.7)_* = 1.07 | .340 | .03 |
| Musical Expertise × Task | *F_(1,32)_* = 0.75 | .395 | .02 |
| Sonic Condition × Task | *F_(2,64)_* = 2.28 | .111 | .07 |
| Musical Expertise × Sonic Condition × Task | *F_(2,64)_* = 0.60 | .552 | .02 |
|  |  |  |  |
| Task Easiness |  |  |  |
| Musical Expertise | *F_(1,32)_* = 0.18 | .678 | < .01 |
| Sonic Condition | *F_(1.6,52.5)_* = 3.01 | .067 | .09 |
| Task | *F_(1,32)_* = 0.97 | .332 | .03 |
| Musical Expertise × Sonic Condition | *F_(1.6,52.5)_* = 0.45 | .604 | .01 |
| Musical Expertise × Task | *F_(1,32)_* = 0.35 | .556 | .01 |
| Sonic Condition × Task | *F_(2,64)_* = 0.13 | .875 | < .01 |
| Musical Expertise × Sonic Condition × Task | *F_(2,64)_* = 0.75 | .475 | .02 |
|  |  |  |  |
| Task Pleasantness |  |  |  |
| Musical Expertise | *F_(1,32)_* = 0.42 | .524 | .01 |
| **Sonic Condition** | ***F_(2,64)_* = 6.35** | **.003**** | **.17** |
| Task | *F_(1,32)_* = 2.93 | .096 | .08 |
| Musical Expertise × Sonic Condition | *F_(2,64)_* = 0.67 | .518 | .02 |
| Musical Expertise × Task | *F_(1,32)_* = 0.49 | .488 | .02 |
| Sonic Condition × Task | *F_(2,64)_* = 0.22 | .805 | < .01 |
| Musical Expertise × Sonic Condition × Task | *F_(2,64)_* = 2.00 | .143 | .06 |

*Note.* Accuracy, percentage of correct words produced, parasite thoughts, task easiness and task pleasantness were analyzed using non-parametric ANOVAs. All other variables were submitted to parametric ANOVAs. * = *p* <.05, ** = *p* <.01, *** = *p* ≤.001.

## Supplementary Table S11

*Quadratic and Cubic Models of Inter-Word Delay in the Verbal Fluency Task, Considering Musical Expertise*

| Effect | Sonic Condition | | | | | |
| --- | --- | --- | --- | --- | --- | --- |
|  | Silence | | Low-arousing | | High-arousing | |
|  | Non Musicians | Musicians | Non Musicians | Musicians | Non Musicians | Musicians |
| R^2^ Linear Model | 0.31 | 0.14 | 0.36 | 0.01 | 0.36 | 0.02 |
| R^2^ Quadratic Model | 0.40 | 0.52 | 0.57 | 0.46 | 0.51 | 0.55 |
| R^2^ Cubic Model | 0.46 | 0.60 | 0.57 | 0.53 | 0.71 | 0.57 |
|  |  |  |  |  |  |  |
| Quadratic Model |  |  |  |  |  |  |
| Linear effect | *F_(1,33)_* = 17.24  *p < .*001*** | *F_(1,29)_* = 8.35  *p = .*007** | *F_(1,29)_* = 24.75  *p < .*001*** | *F_(1,31)_* = 0.51  *p* = .483 | *F_(1,28)_* = 20.62  *p < .*001*** | *F_(1,27)_* = 1.24  *p = .*275 |
| Quadratic effect | *F_(1,33)_* = 4.78  *p = .*036* | *F_(1,29)_* = 23.23  *p < .*001*** | *F_(1,29)_* = 14.27  *p < .*001*** | *F_(1,31)_* = 26.34  *p < .*001*** | *F_(1,28)_* = 8.75  *p = .*006** | *F_(1,27)_* = 32.28  *p < .*001*** |
|  |  |  |  |  |  |  |
| Cubic Model |  |  |  |  |  |  |
| Linear effect | *F_(1,32)_* = 18.54  *p < .*001*** | *F_(1,28)_* = 9.60  *p = .*004** | *F_(1,28)_* = 23.89  *p <.* 001*** | *F_(1,30)_* = 0.56  *p = .*461 | *F_(1,27)_* = 24.47  *p < .*001*** | *F_(1,26)_* = 1.23  *p = .*278 |
| Quadratic effect | *F_(1,32)_* = 5.14  *p = .*030* | *F_(1,28)_* = 26.72  *p < .*001*** | *F_(1,28)_* = 13.78  *p < .*001*** | *F_(1,30)_* = 29.12  *p < .*001*** | *F_(1,27)_* = 10.39  *p* *= .*003** | *F_(1,26)_* = 31.98  *p < .*001*** |
| Cubic effect | *F_(1,32)_* = 3.50  *p = .*071 | *F_(1,28)_* = 5.36  *p = .*028* | *F_(1,28)_* < 0.01  *p =* .975 | *F_(1,30)_* = 4.26  *p = .*047* | *F_(1,27)_* = 6.23  *p = .*019* | *F_(1,26)_* = 0.75  *p = .*394 |

*Note.* *p* < .05 *, *p* < .01**, *p* ≤ .001***.

## Supplementary Analysis 3: Exploratory analysis of Gender Effects

As a further complementary analysis, we tested whether the observed effects were modulated by gender. Our sample was evenly split between females and males (50% each). Below are reported only the significant effects of musical expertise. All other effects are reported in Supplementary Table S10.

**Inter-word delay.** In the VFT, inter-word delays were best predicted by quadratic models in all sonic conditions for both females and males, suggesting an improvement of word retrieval process across time for whatever sonic condition. Supplementary Table S11 reports *R*², coefficients and significance tests details.

**Task easiness.** Concerning tasks execution experience, results revealed a significant Gender × Sonic Condition interaction effect on perceived task easiness. Specifically, females rated tasks execution as easier in the low- (*M* = 12.97, SD = 3.62) than the silence (*M* = 10.85, SD = 3.81; *Z* = 115, *p_adj_* = .031, *r* = .44) and high-arousing conditions (*M* = 11.21, SD = 3.15; *Z* = 343, *p_adj_* = .020, *r* = .47), with no significant difference between the latter two (*Z* = 165, *p_adj_* = .563, *r* = .15). Differently, males considered tasks execution easier when in silence (*M* = 12.15, SD = 3.69) than in the presence of the high-arousing excerpt (*M* = 9.35, SD = 3.38; *Z* = 360, *p* = .027, *r* = .43). No significant differences emerged between the silence and low-arousing conditions (*M* = 11.06, SD = 3.58; *Z* = 268, *p* = .279, *r* = .16), nor between the low- and high-arousing ones (*Z* = 285, *p* = .129, *r* = .33).

Taken together, results do not allow to conclude that background music’ effects depend on participants’ gender, which is in line with previous findings reported in meta-analysis (Cheah et al., 2022). The only notable findings indicate that female found tasks easier to perform in the low-arousing condition over the two other conditions, while males preferred silence over the high-arousing background music.

## Supplementary Table S12

*Inferential Statistics for Each Dependent Variable, Including Gender as Between-Subject Factor*

| Effect | Statistic | *p* | *η_p_^2^* |
| --- | --- | --- | --- |
| *Network Scores* |  |  |  |
| Alert |  |  |  |
| Gender | *F_(1,32)_* = 0.13 | .724 | < .01 |
| Sonic Condition | *F_(2,64)_* = 0.66 | .523 | .02 |
| Gender × Sonic Condition | *F_(2,64)_* = 3.06 | .054 | .09 |
| Orientation |  |  |  |
| Gender | *F_(1,32)_* = 0.02 | .902 | < .01 |
| Sonic Condition | *F_(2,64)_* = 0.13 | .882 | < .01 |
| Gender × Sonic Condition | *F_(2,64)_* = 0.46 | .631 | .01 |
| Executive Control |  |  |  |
| Gender | *F_(1,32)_* = 0.08 | .780 | < .01 |
| **Sonic Condition** | ***F_(2,64)_* = 6.92** | **.002*** | **.18** |
| Gender × Sonic Condition | *F_(2,64)_* = 1.68 | .195 | .05 |
|  |  |  |  |
| *Reaction Times* |  |  |  |
| Gender | *F_(1,32)_* = 2.39 | .132 | .07 |
| **Flanker Type** | ***F_(1,32)_* = 609.93** | **<.001***** | **.95** |
| **Cue Type** | ***F_(1.8,56.6)_* = 65.51** | **<.001***** | **.67** |
| Sonic Condition | *F_(2,64)_* = 0.53 | .590 | .02 |
| Gender × Flanker Type | *F_(1,32)_* = 0.09 | .765 | < .01 |
| Gender × Cue Type | *F_(1.8,56.6)_* = 0.13 | .859 | < .01 |
| Gender × Sonic Condition | *F_(2,64)_* = 0.96 | .390 | .03 |
| **Flanker Type × Cue Type** | ***F_(3,96)_* = 10.04** | **<.001***** | **.24** |
| **Flanker Type × Sonic Condition** | ***F_(2,64)_* = 6.51** | **.003**** | **.17** |
| Cue Type × Sonic Condition | *F_(6,192)_* = 0.66 | .683 | .02 |
| Gender × Flanker Type × Cue Type | *F_(3,96)_* = 0.19 | .904 | < .01 |
| Gender × Flanker Type × Sonic Condition | *F_(2,64)_* = 1.27 | .287 | .04 |
| Gender × Cue Type × Sonic Condition | *F_(6,192)_* = 0.86 | .523 | .03 |
| Flanker Type × Cue Type × Sonic Condition | *F_(6,192)_* = 0.80 | .569 | .02 |
| Gender × Flanker Type × Cue Type × Sonic Condition | *F_(6,192)_* = 0.18 | .981 | < .01 |
|  |  |  |  |
| *Accuracy* |  |  |  |
| Gender | *F_(1,32)_* = 0.05 | .831 | < .01 |
| **Flanker Type** | ***F_(1,32)_* = 280.96** | **<.001***** | **.90** |
| **Cue Type** | ***F_(3,96)_* = 5.20** | **.002**** | **.14** |
| Sonic Condition | *F_(2,64)_* = 0.43 | .651 | .01 |
| Gender × Flanker Type | *F_(1,32)_* = 3.07 | .089 | .09 |
| Gender × Cue Type | *F_(3,96)_* = 1.50 | .221 | .05 |
| Gender × Sonic Condition | *F_(2,64)_* = 0.23 | .798 | < .01 |
| **Flanker Type × Cue Type** | ***F_(3,96)_* = 8.17** | **<.001***** | **.20** |
| Flanker Type × Sonic Condition | *F_(2,64)_* = 0.97 | .386 | .03 |
| Cue Type × Sonic Condition | *F_(6,192)_* = 1.13 | .346 | .03 |
| Gender × Flanker Type × Cue Type | *F_(3,96)_* = 0.86 | .465 | .03 |
| Gender × Flanker Type × Sonic Condition | *F_(2,64)_* = 0.19 | .830 | < .01 |
| Gender × Cue Type × Sonic Condition | *F_(6,192)_* = 1.80 | .100 | .05 |
| Flanker Type × Cue Type × Sonic Condition | *F_(6,192)_* = 1.06 | .390 | .03 |
| Gender × Flanker Type × Cue Type × Sonic Condition | *F_(6,192)_* = 1.82 | .553 | .03 |
|  |  |  |  |
| *Percentage of Correct Words Produced* |  |  |  |
| Gender | *F_(1,32)_* = 0.13 | .718 | <.01 |
| Sonic Condition | *F_(2,64)_* = 022 | .804 | <.01 |
| Gender × Sonic Condition | *F_(2,64)_* = 1.11 | .336 | .03 |
|  |  |  |  |
| *NASA-TLX – Global Score* |  |  |  |
| Gender | *F_(1,32)_* < 0.01 | 1.000 | < .01 |
| **Sonic Condition** | ***F_(2,64)_* = 3.90** | **.025*** | **.11** |
| **Task** | ***F_(1,32)_* = 4.24** | **.048** | **.12** |
| Gender × Sonic Condition | *F_(2,64)_* = 0.71 | .494 | .02 |
| Gender × Task | *F_(1,32)_* = 0.06 | .813 | < .01 |
| **Sonic Condition × Task** | ***F_(2,64)_* = 3.70** | **.030*** | **.10** |
| Gender × Sonic Condition × Task | *F_(2,64)_* = 1.94 | .152 | .06 |
|  |  |  |  |
| *Subjective Experience* |  |  |  |
| Parasite Thoughts |  |  |  |
| Gender | *F_(1,32)_* = 1.66 | .207 | .05 |
| Sonic Condition | *F_(2,64)_* = 2.27 | .112 | .07 |
| **Task** | ***F_(1,32)_* = 15.28** | **.001***** | **.32** |
| Gender × Sonic Condition | *F_(2,64)_* = 1.56 | .217 | .05 |
| Gender × Task | *F_(1,32)_* = 1.81 | .188 | .05 |
| Sonic Condition × Task | *F_(2,64)_* = 2.10 | .131 | .06 |
| Gender × Sonic Condition × Task | *F_(2,64)_* < 0.01 | .994 | <.01 |
|  |  |  |  |
| Task Easiness |  |  |  |
| Gender | *F_(1,32)_* = 0.84 | .185 | .05 |
| Sonic Condition | *F_(2,64)_* = 2.89 | .063 | .08 |
| Task | *F_(1,32)_* = 0.74 | .398 | .02 |
| **Gender × Sonic Condition** | ***F_(2,64)_* = 3.67** | **.031*** | **.10** |
| Gender × Task | *F_(1,32)_* = 2.75 | .107 | .08 |
| Sonic Condition × Task | *F_(2,64)_* = 0.46 | .634 | .01 |
| Gender × Sonic Condition × Task | *F_(2,64)_* = 0.93 | .401 | .03 |
|  |  |  |  |
| Task Pleasantness |  |  |  |
| Gender | *F_(1,32)_* = 0.73 | .400 | .02 |
| **Sonic Condition** | ***F_(2,64)_* = 9.70** | **<.001***** | **.23** |
| Task | *F_(1,32)_* = 2.50 | .123 | .07 |
| Gender × Sonic Condition | *F_(2,64)_* = 3.04 | .055 | .09 |
| Gender × Task | *F_(1,32)_* = 1.18 | .286 | .04 |
| Sonic Condition × Task | *F_(2,64)_* = 1.05 | .355 | .03 |
| Gender × Sonic Condition × Task | *F_(2,64)_* = 1.82 | .171 | .05 |

*Note.* Accuracy, percentage of correct words produced, parasite thoughts, task easiness and task pleasantness were analyzed using non-parametric ANOVAs. All other variables were submitted to parametric ANOVAs. *p* < .05 *, *p* < .01**, *p* ≤ .001***.

## Supplementary Table S13

*Quadratic and Cubic Models of Inter-Word Delay in the Verbal Fluency Task, Considering Gender*

| Effect | Sonic Condition | | | | | |
| --- | --- | --- | --- | --- | --- | --- |
|  | Silence | | Low-arousing | | High-arousing | |
|  | Females | Males | Females | Males | Females | Males |
| R^2^ Linear Model | 0.03 | 0.47 | 0.36 | 0.16 | 0.08 | 0.29 |
| R^2^ Quadratic Model | 0.45 | 0.56 | 0.51 | 0.58 | 0.55 | 0.43 |
| R^2^ Cubic Model | 0.48 | 0.61 | 0.52 | 0.58 | 0.55 | 0.57 |
|  |  |  |  |  |  |  |
| Quadratic Model |  |  |  |  |  |  |
| Linear effect | *F_(1,27)_* = 1.33  *P* = .259 | *F_(1,33)_* = 35.37  *p < .*001*** | *F_(1,26)_* = 19.00  *p < .*001*** | *F_(1,32)_* = 12.14  *p* = .002** | *F_(1,28)_* = 4.69  *p = .*039* | *F_(1,26)_* = 12.89  *p* = .001** |
| Quadratic effect | *F_(1,27)_* = 21.09  *p < .*001*** | *F_(1,33)_* = 7.20  *p = .*011* | *F_(1,26)_* = 7.65  *p = .*010* | *F_(1,32)_* = 31.35  *p < .*001*** | *F_(1,28)_* = 28.80  *p < .*001*** | *F_(1,26)_* = 6.34  *p = .*018* |
|  |  |  |  |  |  |  |
| Cubic Model |  |  |  |  |  |  |
| Linear effect | *F_(1,26)_* = 1.36  *p = .*255 | *F_(1,32)_* = 38.65  *p < .*001*** | *F_(1,25)_* = 18.59  *p <.* 001*** | *F_(1,31)_* = 11.76  *p = .*002** | *F_(1,27)_* = 4.60  *p = .*041* | *F_(1,25)_* = 12.58  *p = .*002** |
| Quadratic effect | *F_(1,26)_* = 21.52  *p < .*001*** | *F_(1,32)_* = 7.87  *p = .*008** | *F_(1,25)_* = 7.49  *p = .*011* | *F_(1,31)_* = 30.38  *p < .*001*** | *F_(1,27)_* = 28.27  *p* < *.*001*** | *F_(1,25)_* = 6.19  *p = .*020* |
| Cubic effect | *F_(1,26)_* = 1.55  *p = .*225 | *F_(1,32)_* = 4.06  *p = .*052 | *F_(1,25)_* = 0.44  *p =* .513 | *F_(1,31)_* = 0.01  *p = .*927 | *F_(1,27)_* = 0.49  *p = .*491 | *F_(1,25)_* = 0.37  *p = .*551 |

*Note.* *p* < .05 *, *p* < .01**, *p* ≤ .001***.

**Supplementary References**

Alluri, V., Toiviainen, P., Jääskeläinen, I. P., Glerean, E., Sams, M., & Brattico, E. (2012). Large-scale brain networks emerge from dynamic processing of musical timbre, key and rhythm. *NeuroImage*, *59*(4), 3677–3689. https://doi.org/10.1016/j.neuroimage.2011.11.019

Cheah, Y., Wong, H. K., Spitzer, M., & Coutinho, E. (2022). Background Music and Cognitive Task Performance: A Systematic Review of Task, Music, and Population Impact. *Music and Science*, *5*, 1–44. https://doi.org/10.1177/20592043221134392

Fernandez, N. B., Trost, W. J., & Vuilleumier, P. (2019). Brain networks mediating the influence of background music on selective attention. *Social Cognitive and Affective Neuroscience*, *14*(12), 1441–1452. https://doi.org/10.1093/scan/nsaa004

Gomez, P., & Danuser, B. (2007). Relationships between musical structure and psychophysiological measures of emotion. *Emotion*, *7*(2), 377–387. https://doi.org/10.1037/1528-3542.7.2.377

Lartillot, O., & Grandjean, D. (2019). Tempo and metrical analysis by tracking multiple metrical levels using autocorrelation. *Applied Sciences*, *9*(23). https://doi.org/10.3390/app9235121

Lartillot, O., Toiviainen, P., & Eerola, T. (2008). A matlab toolbox for music information retrieval. In *Data Analysis, Machine Learning and Applications: Proceedings of the 31st Annual Conference of the Gesellschaft für Klassifikation eV, Albert-Ludwigs-Universität Freiburg, March 7–9*. Springer Berlin Heidelberg. https://doi.org/10.1007/978-3-540-78246-9_31

Patston, L. L., & Tippett, L. J. (2011). The effect of background music on cognitive performance in musicians and nonmusicians. *Music Perception*, *29*(2), 173–183. https://doi.org/https://doi.org/10.1525/mp.2011.29.2.173

Schubert, E. (1999). Measurement and Time Series Analysis of Emotion in Music [UNSW Sydney]. In *Department of Philosophy*. https://doi.org/10.26190/unsworks/6566

Trost, W., Frühholz, S., Cochrane, T., Cojan, Y., & Vuilleumier, P. (2015). Temporal dynamics of musical emotions examined through intersubject synchrony of brain activity. *Social Cognitive and Affective Neuroscience*, *10*(12), 1705–1721. https://doi.org/10.1093/scan/nsv060

Tzanetakis, G., & Cook, P. (2010). Musical genre classification of audio signals using geometric methods. *European Signal Processing Conference*, *10*(5), 497–501.
